# Additives Altered Bacterial Communities and Metabolic Profiles in Silage Hybrid *Pennisetum*

**DOI:** 10.3389/fmicb.2021.770728

**Published:** 2022-01-05

**Authors:** Hanchen Tian, Yanchen Zhu, Mengxue Dai, Tong Li, Yongqing Guo, Ming Deng, Baoli Sun

**Affiliations:** College of Animal Science, South China Agricultural University, Guangzhou, China

**Keywords:** hybrid *Pennisetum*, silage additives, bacterial community, metabolic profile, silage quality

## Abstract

This study was conducted to investigate the effects of different additives on the fermentation quality, nutrient composition, bacterial communities, and metabolic profiles of the silage of hybrid *Pennisetum*. The experiment was conducted using five treatments, i.e., CK, control group, MA, 1% malic acid of fresh matter (FM) basis, GL, 1% glucose of FM basis, CE, 100 U/g FM cellulase, and BS, 10^6^ cfu/g FM *Bacillus subtilis*, with six replicates each treatment. After a 120-day fermentation, 30 silage packages were opened for subsequent determination. As a result, all four additives had positive effects on the fermentation quality and nutrient composition of the silage of hybrid *Pennisetum*. The high-throughput sequencing of V3–V4 regions in 16S rRNA was performed, and results showed that *Firmicutes* and *Proteobacteria* were the dominant phyla and that *Aquabacterium* and *Bacillus* were the dominant genera. MA, GL, CE, and BS treatment resulted in 129, 21, 25, and 40 differential bacteria, respectively. The four additives upregulated *Bacillus smithii* but downregulated *Lactobacillus rossiae*. Metabolic profiles were determined by UHPLC-Q/TOF-MS technology and the differential metabolites caused by the four additives were 47, 13, 47, and 18, respectively. These metabolites played antioxidant, antibacterial, and anti-inflammatory functions and involved in pathways, such as the citrate cycle, carbon fixation in photosynthetic organisms, and glyoxylate and dicarboxylate metabolism. In conclusion, silage additives promoted fermentation quality and nutrient composition by altering bacterial communities and metabolic profiles. This study provided potential biomarkers for the improvement of silage quality.

## Introduction

The hybrid *Pennisetum* (*Pennisetum americanum* × *Pennisetum purpureum*), a kind of high-stalk perennial plant belonging to the Poaceae family, is a fast-growing and high-yielding agricultural crop widely distributed in south China (Song et al., 2019; Cai et al., 2020). With strong adaptability, the hybrid *Pennisetum* requires minimum management and can resist adverse conditions, including drought, flood, acid, and salt, and can grow on barren land due to its vigorous root system (Wang Y. et al., 2019). The hybrid *Pennisetum* has a wide range of uses and has been reported for use as feedstock for biogas and biofuel and as forage and ornamental plant (Wang et al., 2014; Suaisom et al., 2019; Zhao et al., 2019).

Ensiling is an effective and widespread technique for long-term feed preservation with the characteristics of low cost and easy operation (Wang et al., 2021). At the early stage of ensiling, water-soluble carbohydrates (WSC) are broken down into water, carbon dioxide, and energy under the respiration of aerobic bacteria (Zieliński et al., 2021). When oxygen is depleted, lactic acid bacteria (LAB) attached to forage multiply and convert WSC into organic acids, thereby creating an acidic and anaerobic environment, inhibiting the activities of unwanted bacteria, such as clostridia, and reducing the risk of forage spoilage (Zieliński et al., 2021). By 2050, more than half of the global demand for ruminant meat and milk is estimated to be produced in developing countries particularly in China and India (Xu et al., 2021). Silage is an important part of ruminant feed, and its safety and quality become a requirement for the steady development of ruminant husbandry to a certain extent. The hybrid *Pennisetum* is recognized as a forage grass with high buffer capacity and low WSC content and is difficult to ensile (Shah et al., 2020b). Therefore, silage additives, including chemicals, enzymes, and LAB and non-LAB species, appear to be important as they play important roles in improving lactate fermentation, suppressing spoilage microorganisms, enhancing aerobic stability, and reducing nutrient degradation (Muck et al., 2018).

The silage quality is attributed to the bacterial community. Types and breeds of forage, temperature of ensiling, length of storage, and application of silage additives have remarkable effects on the composition and function of bacterial community (Carvalho-Estrada et al., 2020). However, due to the complex composition of microbial community, traditional microbial isolation and culture technologies, including culture-dependent strain isolation and microorganism counting, cannot be adapted for the exploration of dynamic changes in silage microflora (Guan et al., 2020). Recently, the wide application of high-throughput sequencing technology, including Illumina MiSeq, Ion Torrent PGM, single-molecule real-time sequencing technology, and 454 sequencing FLX Titanium chemistry, provides new insights into the microbial ecology of silage (McAllister et al., 2018). With the process of fermentation, the activities of microorganisms produce a large number of metabolites, including amino acids, aromatic compounds, fatty acids, flavoring agents, oligosaccharides, peptides, and vitamins (Guo et al., 2018). In addition to lactic acid (LA), acetic acid, propionic acid, and other conventionally detected substances, other metabolites, such as sorbic acid, isovaleric acid, 3-phenyllactic acid, and hydroferulic acid, play important roles in maintaining aerobic stability and improving the fermentation quality of silage (Hu et al., 2020). Metabolomics focuses on all small-molecule metabolites with molecular weights less than 1,000 Da and can monitor the changes in metabolic profiles caused by different treatments. Emerging microbiome and metabolome technologies provide new insights for silage research. The combined analyses of microbiome and metabolome may reveal potential biological processes during ensiling (Wang et al., 2020). In recent years, Napier grass, alfalfa, sainfoin, whole-crop corn, rice straw, and stylo silage have been subjected to multiomics analyses. These previous studies revealed the mechanism of different additives affecting silage quality to a certain extent (Guan et al., 2020; Wang et al., 2020; Xu et al., 2020, 2021; Zhang et al., 2021).

To our knowledge, the combined analysis of microbiome and metabolome on hybrid *Pennisetum* silage (HPS) remains limited. We hypothesize that silage additives have different effects and corresponding mechanisms on silage quality. Therefore, in this study, HPS is treated with four types of additives, i.e., malic acid (fermentation inhibitor), glucose (fermentation accelerator), cellulase (enzyme), and *Bacillus subtilis* (microbial inoculant). After a 120-day fermentation, the fermentation parameters, chemical compositions, bacterial community, and metabolic profile of HPS are determined. In addition, the combined analyses of microbiome and metabolome are performed. This study may reveal the potential mechanisms of different additives affecting silage quality and provide theoretical reference for the safe production of silage.

## Materials and Methods

### Silage Preparation

The fresh hybrid *Pennisetum* was harvested from a commercial plantation base located in Meizhou City, Guangdong Province (115.82°E, 24.52°N). Plants were cut down at a height of 2–2.5 m and chopped into pieces (1–2 cm per segment) *via* a fully automatic grass shredding machine. The chemical composition of hybrid *Pennisetum* was determined (Table 1). After moderate drying, the chopped hybrid *Pennisetum* was divided into five treatments and ensiled with (1) no additive (CK), (2) 1% malic acid (purity ≥ 99.5%; Shanghai Macklin Biochemical Co., Ltd., Shanghai) of fresh matter (MA), (3) 1% glucose (purity ≥ 99.5%; Shanghai Macklin Biochemical) of fresh matter (GL), (4) 100 U/g cellulase (VTR Bio-Tech Co., Ltd., Zhuhai, Guangdong) of fresh matter (CE), and (5) 10^6^ cfu/g *Bacillus subtilis* (VTR Bio-Tech) of fresh matter (BS). After adequate mixing, approximately 200 g hybrid *Pennisetum* was packed into a polyethylene bag (20 cm × 30 cm), compacted, and sealed *via* an automatic vacuum packager. Six replicates were set in a treatment; 30 bags were obtained and preserved at ambient temperature (25–28°C). After a 120-day fermentation, all 30 packages were opened for subsequent experiments, including the determination of fermentation parameters and chemical compositions and the analyses of bacterial community and metabolic profile.

**TABLE 1:** Chemical compositions of hybrid *Pennisetum.*

Items	Content
DM (g/kg FM)	336.30
WSC (g/kg DM)	26.84
Starch (g/kg DM)	8.78
NDF (g/kg DM)	696.50
ADF (g/kg DM)	431.72
CP (g/kg DM)	68.05
TP (g/kg TN)	742.91
NPN (g/kg TN)	257.09
NDIN (g/kg TN)	252.61
ADIN (g/kg TN)	188.91

*DM, dry matter; FM, fresh matter; WSC, water-soluble carbohydrates; NDF, neutral detergent fiber; ADF, acid detergent fiber; CP, crude protein; TN, total protein; TP, true protein; NPN, non-protein nitrogen; NDIN, neutral detergent insoluble nitrogen; ADIN, acid detergent insoluble nitrogen.*

### Determination of Fermentation Parameters and Chemical Compositions

Approximately 4 g of each silage sample was divided into two parts and maintained at −80°C for the determination of bacterial community and metabolic profile. Then, 10 g sample was homogenized with 90 ml distilled water and incubated at 4°C for 24 h. After extraction, the mixture was filtered by a sterilized 4-layer gauze, and the filtrate was collected to measure fermentation parameters. pH was determined using a glass electrode pH meter (FE28-Standard); volatile fatty acids (VFAs) and LA were determined in accordance with the method of Rumsey et al. (1967); and ammoniacal nitrogen (AN) was measured *via* phenol–hypochlorite colorimetry (Zhang et al., 2021).

The rest of the silage samples was dried to constant weight at 65°C for 48 h for the determination of dry matter (DM). Then, the dried sample was ground for the analyses of chemical compositions. WSC and crude protein (CP) contents were determined in accordance with the procedure of the Association of Official Analytical Chemists (AOAC, 2002). Neutral (NDF) and acid (ADF) detergent fibers were measured in accordance with the methods described by Van Soest et al. (1991). True protein (TP) and non-protein nitrogen (NPN) contents were determined using the trichloroacetic acid method (Licitra et al., 1996). Neutral (NDIN) and acid (ADIN) detergent insoluble nitrogen contents were measured in accordance with the method of Licitra et al. (1996). The content of starch was analyzed *via* perchloric acid–anthrone colorimetry reported by Bakhshy et al. (2020).

### Bacterial Community Analyses

The total genomic DNA of silage sample was isolated *via* the DNeasy Power Soil Kit (QIAGEN, Inc., Netherlands) on the basis of the manufacturer’s instructions. After isolation, the concentration and quality of DNA samples were determined using the NanoDrop2000 Spectrophotometer (Thermo, United States). The Pyrobest DNA Polymerase (TaKaRa, DR500A) was adopted for the amplification of 16S rRNA V3–V4 regions of genomic DNA, and primer pairs were designed as 338F (5′-ACTCCTACGGGAGGCA GCA-3′) and 806R (5′-GACTACHVGGGTATCTAATCC-3′) in accordance with the method of Zi et al. (2021). The quality control and purification of PCR products were subsequently performed after amplification *via* the PicoGreen dsDNA Assay Kit (Invitrogen, Carlsbad, CA, United States) and Agencourt AMPure Beads (Beckman Coulter, Indianapolis, IN, United States). The high-throughput sequencing was carried out by the equimolar and paired-end sequencing (PE250) on the Illumina Novaseq 6000 platform (Personal Biotechnology Co., Ltd., Shanghai, China).

After sequencing, the processing of sequenced data was implemented by the QIIME (V 1.8.0) software. After the filtration of low-quality sequences defined by Gill et al. (2006) and Chen and Jiang (2014) and meaningless sequences (Including adapters, chimera, poly_A and primer), valid sequences were clustered into operational taxonomic units (OTUs) at a 97% similarity threshold *via* the UCLUST (Malik et al., 2020). The Basic Local Alignment Search Tool was used for further taxonomic classification, and an OTU table was obtained (Urbanek et al., 2020). The α- and β-diversity values of bacterial community were calculated *via* the QIIME software and vegan package in R software, respectively (De Filippis et al., 2018). The functions of bacterial community were predicted *via* the Phylogenetic Investigation of Communities by Reconstruction of Unobserved States (PICRUSt) database (Langille et al., 2013). The Linear Discriminant Analysis (LDA) Effect Size (LEfSe) analyses were conducted *via* an online tool^1^, and LDA score>3 and *p* < 0.05 were selected as threshold.

### Metabolic Profile Analyses

In this study, the metabolic profile was analyzed *via* the UHPLC-Q/TOF-MS technology. The thawed sample (100 mg) was ground promptly and homogenized in 1 ml precooled methanol/acetonitrile/ddH_2_O solvent (2:2:1, v/v/v), and the mixture was subjected to cryogenic ultrasound at −20°C for 30 min. After standing for 10 min, the mixture was centrifuged at 14,000 rpm and 4°C for 15 min. The supernatant was reserved and dried by a vacuum centrifuge, and the dried sample was redissolved and homogenized in 100 μl acetonitrile/ddH_2_O solvent (1:1, v/v). After centrifugation at 14,000 rpm and 4°C for 15 min, the supernatant was collected for analyses. In addition, quality control (QC) samples were prepared to monitor the repeatability and stability of instruments.

Samples were analyzed using the Agilent 1290 Infinity LC UHPLC system and the parameters of instrument were set as follows: column temperature, 25°C; flow velocity, 0.5 ml/min; injection volume, 2 μl; mobile phases, and 25 mM ammonium acetate + 25 mM ammonium hydroxide in ddH_2_O (A) and acetonitrile (B). The elution procedure was as follows: 0–0.5 min, B maintained at 95%; 0.5–7 min, B changed from 95 to 65% linearly; 7–8 min, B changed from 65 to 40% linearly; 8–9 min, B was 40%; 9–9.1 min, B linearly changed from 40 to 95%; 9.1–12 min, B maintained at 95%. Samples were stored in an autosampler at 4°C during the whole analysis period.

The AB Sciex Triple TOF 6600 mass spectrometer was adopted for analyses, and the parameters of electrospray ionization source were set in accordance with the method of Li R. et al. (2021). Briefly, the Ion Source Gas1, Ion Source Gas2, and Curtain gas were 60, 60, and 30 kPa, respectively. The IonSpray Voltage Floating was ±5,500 V for positive and negative modes. The source temperature was 600°C. For MS-only acquisition, the m/z range of the TOF MS scan and product ion scan were 60–1,000 and 25–1,000 Da, respectively. The accumulation time of TOF MS scan and product ion scan were 0.20 and 0.05 s/spectra, respectively. The information-dependent acquisition was adopted for the acquisition of the product ion scan, and parameters were set as follows: mode, high sensitivity; declustering potential, ±60 V for positive and negative modes; collision energy, 35 V ± 15 eV. Ten candidate ions were supervised per cycle, and isotopes within 4 Da were eliminated.

Data processing was performed in accordance with the method of Li R. et al. (2021). First, raw data in the wiff.scan format were converted into the MzXML format *via* the ProteoWizard MSConvert, and MzXML files were imported into the XCMS software. The parameters for peak picking were set as centWave m/z = 25 ppm, prefilter = c (10, 100), and peak width = c (10, 60). bw = 5, minfrac = 0.5, and mzwid = 0.025 were used as parameters for peak grouping. The annotation of adducts and isotopes was performed using the Collection of Algorithms of MEtabolite pRofile Annotation. The structural identification of metabolites was performed by comparing the retention time, molecular weight (<25 ppm), MS/MS spectra, and collision energy with the database established by Benton et al. (2015). Subsequently, multidimensional statistical analyses, including principal component analysis (PCA), projections to latent structures–discriminant analysis (PLS-DA), and orthogonal PLS-DA (OPLS-DA), were conducted using the SIMCA-P 14.1 software. Metabolites with variable importance in projection (VIP) > 1, fold change > 1.2 or <0.833, and *p* < 0.05 were recognized as differential metabolites (Hao et al., 2021). In addition, the Kyoto Encyclopedia of Genes and Genomes (KEGG) database was adopted for the analyses of functional characteristics and classifications of differential metabolites (Guan et al., 2020).

### Statistical Analysis

The experimental data of fermentation parameters, chemical compositions, and α-diversity indices were preliminarily sorted using the Excel software and analyzed *via* the SAS 9.4 software with the model of *Y*_*ij*_ = μ + *A*_*i*_ + ε_*ij*_. *Y*_*ij*_ is the dependent variable of the silage samples in different treatments, μ is the overall mean, A_*i*_ is the effects of silage additives, and ε_*ij*_ is the random error. The LSD method was adopted for multiple comparisons. Data were displayed in tables in the form of mean ± SD, and *p* < 0.05 indicated a significant difference. The Pearson correlation coefficient was adopted for correlation analyses, and correlation coefficient (Cor) > 0.6 or <−0.6 and *p* < 0.05 were considered as correlation.

## Results

### Characteristics of Fresh Hybrid *Pennisetum*

The chemical compositions of the hybrid *Pennisetum* are shown as Table 1. The DM content of hybrid *Pennisetum* was 336.30 g/kg fresh matter (FM). The contents of WSC, starch, NDF, ADF, and CP were 26.84, 8.78, 696.50, 431.72, and 68.05 g/kg DM, respectively. The contents of TP, NPN, NDIN, and ADIN were 742.91, 257.09, 252.61, and 188.91 g/kg total nitrogen (TN), respectively.

### Fermentation Parameters and Chemical Compositions of Hybrid *Pennisetum* Silage

As shown in Table 2, glucose and cellulase increased and decreased, respectively, the DM content in HPS (*p* < 0.05). The application of malic acid and cellulase decreased the pH of HPS (*p* < 0.05). Malic acid increased the content of LA but decreased the AA content (*p* < 0.05). Compared with that in CK, the AA content in CE continued to increase (*p* < 0.05). WSC contents in GL, CE, and BS were significantly lower than those in CK (*p* < 0.05). The contents of AN in MA and CE decreased compared with that in CK (*P* < 0.05). The chemical compositions of HPS are shown in Table 3. Malic acid, cellulase, and *B. subtilis* reduced the contents of NDF in HPS (*p* < 0.05). Moreover, cellulase and *B. subtilis* could reduce the contents of starch and ADF (*p* < 0.05). Compared with CK, GL, CE, and BS were observed with increased CP and significantly decreased NDIN and ADIN (*p* < 0.05). In addition, the application of malic acid and cellulase could increase the content of TP (*p* < 0.05).

**TABLE 2:** Effects of silage additives on the fermentation parameters of silage hybrid *Pennisetum.*

	CK	MA	GL	CE	BS
DM (g/kg FM)	322.49 ± 5.02^bc^	326.74 ± 3.15^ac^	330.40 ± 7.09^a^	320.86 ± 3.06^c^	321.41 ± 1.45^bc^
pH	4.08 ± 0.02^a^	4.03 ± 0.02^b^	4.08 ± 0.02^a^	4.05 ± 0.02^b^	4.10 ± 0.02^a^
LA (g/kg DM)	7.41 ± 0.16^bc^	8.34 ± 0.55^a^	7.13 ± 0.63^c^	8.00 ± 0.51^ab^	7.71 ± 0.50^ab^
AA (g/kg DM)	6.64 ± 0.33^b^	4.86 ± 0.23^c^	6.94 ± 0.30^b^	8.46 ± 0.84^a^	6.83 ± 0.69^b^
WSC (g/kg DM)	23.79 ± 3.96^a^	21.44 ± 2.73^ab^	18.87 ± 2.24^b^	19.80 ± 1.51^b^	18.54 ± 1.65^b^
AN (g/kg TN)	59.05 ± 7.99^a^	36.93 ± 4.61^b^	56.14 ± 2.90^a^	33.93 ± 4.03^b^	57.29 ± 1.77^a^

*CK, control group; MA, 1% FM malic acid addition; GL, 1% FM glucose addition; CE, 100 U/g FM cellulase addition; BS, 10^6^ cfu/g Bacillus subtilis FM addition. DM, dry matter; FM, fresh matter; LA, lactic acid; AA, acetic acid; WSC, water-soluble carbohydrates; AN, ammonia nitrogen; TN, total nitrogen. ^a,b,c^Different letters indicate significant differences (P < 0.05).*

**TABLE 3:** Effects of silage additives on the chemical compositions of silage hybrid *Pennisetum.*

	CK	MA	GL	CE	BS
Starch (g/kg DM)	7.34 ± 0.40^a^	7.19 ± 1.10^a^	6.57 ± 0.85^ab^	5.56 ± 0.56^c^	6.20 ± 0.34^bc^
NDF (g/kg DM)	668.10 ± 16.88^a^	647.37 ± 13.58^b^	665.01 ± 9.48^a^	603.06 ± 12.81^c^	633.93 ± 6.81^b^
ADF (g/kg DM)	414.57 ± 18.94^a^	405.13 ± 8.56^a^	412.03 ± 10.19^a^	367.20 ± 8.77^c^	386.50 ± 6.34^b^
CP (g/kg DM)	56.10 ± 2.06^d^	56.86 ± 1.41^d^	59.73 ± 1.25^c^	72.25 ± 1.48^a^	61.72 ± 1.01^b^
TP (g/kg TN)	637.38 ± 18.00^c^	675.66 ± 24.61^b^	631.21 ± 13.78^c^	716.63 ± 11.05^a^	626.35 ± 12.58^c^
NPN (g/kg TN)	362.62 ± 18.00^a^	324.34 ± 24.61^b^	368.79 ± 13.78^a^	283.37 ± 11.05^c^	373.65 ± 12.58^a^
NDIN (g/kg TN)	284.90 ± 5.16^a^	287.17 ± 7.15^a^	246.11 ± 10.98^b^	194.54 ± 17.68^c^	236.60 ± 5.33^b^
ADIN (g/kg TN)	182.99 ± 7.85^a^	178.19 ± 5.42^a^	158.57 ± 7.98^b^	122.77 ± 7.68^d^	147.03 ± 3.51^c^

*CK, control group; MA, 1% FM malic acid addition; GL, 1% FM glucose addition; CE, 100 U/g FM cellulase addition; BS, 10^6^ cfu/g Bacillus subtilis FM addition. DM, dry matter; FM, fresh matter; NDF, neutral detergent fiber; ADF, acid detergent fiber; CP, crude protein; TN, total protein; TP, true protein; NPN, non-protein nitrogen; NDIN, neutral detergent insoluble nitrogen; ADIN, acid detergent insoluble nitrogen. ^a,b,c^Different letters indicate significant differences (P < 0.05).*

### Bacterial Diversity in Hybrid *Pennisetum* Silage Affected by Silage Additives

**FIGURE 1:**
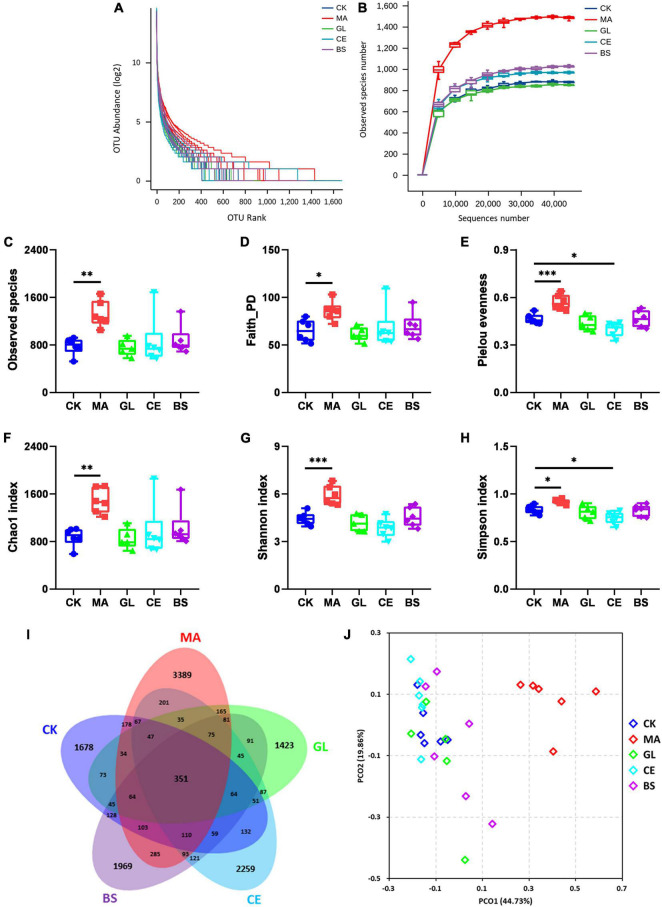
Effects of silage additives on the diversity of bacterial communities in hybrid *Pennisetum* silage. **(A)** Rank–abundance curve; **(B)** rarefaction curve; **(C)** observed species index; **(D)** Faith PD index; **(E)** Pielou evenness index; **(F)** Chao1 index; **(G)** Shannon index; **(H)** Simpson index; **(I)** venn diagram of operational taxonomic units (OTUs); **(J)** principal co-ordinates analysis (PCoA) scores plot. CK, control group; MA, 1% fresh matter (FM) malic acid addition; GL, 1% FM glucose addition; CE, 100 U/g FM cellulase addition; BS, 10^6^ cfu/g *Bacillus subtilis* FM addition. *, **, and *** represent *P* < 0.05, *P* < 0.01, and *P* < 0.001, respectively.

In this study, silage samples were sequenced *via* the Illumina Novaseq 6000 platform, and 2,548,477 raw reads were obtained. After removing meaningless sequences, we obtained 2,011,503 clean sequences. The clustering and annotation of clean reads were performed, and 13,503 OTUs were found eventually. The diversity of bacterial communities is shown in Figure 1. The rank–abundance curve showed that samples in MA had improved evenness and diversity (Figure 1A). With increasing sequence number, the rarefaction curve tended to flatten, indicating that sampling was sufficient and that data were reasonable and reliable for further analyses. For α-diversity (Figures 1C–H and Supplementary Table 1), the Observed species, Faith PD, Pielou evenness, Chao1, Shannon, and Simpson indices of MA were significantly higher than those in CK (*p* < 0.05). Moreover, the Pielou evenness and Simpson indices in CE were lower compared with those in CK (*p* < 0.05). The Venn diagram showed that 351 OTUs appeared in all five treatments, and 1,678, 3,389, 1,423, 2,259, and 1,969 OTUs were unique to CK, MA, GL, CE, and BS, respectively (Figure 1I). For β-diversity, the PCoA score plot indicated that the individuals in MA could be remarkably separated from those in four other treatments. The Adonis analyses were further conducted, and results showed that the application of malic acid, cellulase, and *B. subtilis* had significant effects on the bacterial communities in HPS (*p* < 0.05). However, no evident difference between CK and GL was observed (*p* < 0.05).

### Bacterial Abundance in Hybrid *Pennisetum* Silage Affected by Silage Additives

**FIGURE 2:**
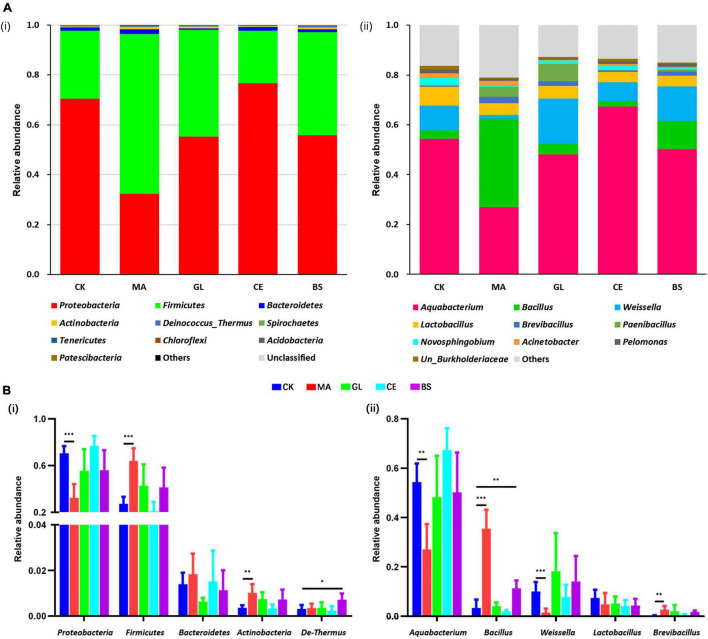
Relative abundance of bacterial communities at the phylum and genus levels for hybrid *Pennisetum* silage treated with different silage additive. **(A)** Accumulation bar graph of the top 10 phyla **(i)** and genera **(ii)**. **(B)** Bar graph of the top five phyla **(i)** and genera **(ii)**. CK, control group; MA, 1% fresh matter (FM) malic acid addition; GL, 1% FM glucose addition; CE, 100 U/g FM cellulase addition; BS, 10^6^ cfu/g *Bacillus subtilis* FM addition. *, **, and *** represent *P* < 0.05, *P* < 0.01, and *P* < 0.001, respectively.

At the phylum level, the dominant bacteria were *Proteobacteria*, *Firmicutes*, *Bacteroidetes*, *Actinobacteria*, and *Deinococcus–Thermus* (Figure 2A and Supplementary Table 2). Malic acid reduced the relative abundance of *Proteobacteria* but increased *Firmicutes* and *Actinobacteria* (*p* < 0.05). The addition of *B. subtilis* increased the abundance of *Deinococcus–Thermus* (*p* < 0.05, Figure 2B and Supplementary Table 2). At the genus level, the dominant bacteria were *Aquabacterium*, *Bacillus*, *Weissella*, *Lactobacillus*, and *Brevibacillus* (Figure 2A and Supplementary Table 2). The application of malic acid decreased the abundance of *Aquabacterium* and *Weissella* but increased the abundance of *Bacillus* and *Brevibacillus* (*p* < 0.05, Figure 2B and Supplementary Table 2).

**FIGURE 3:**
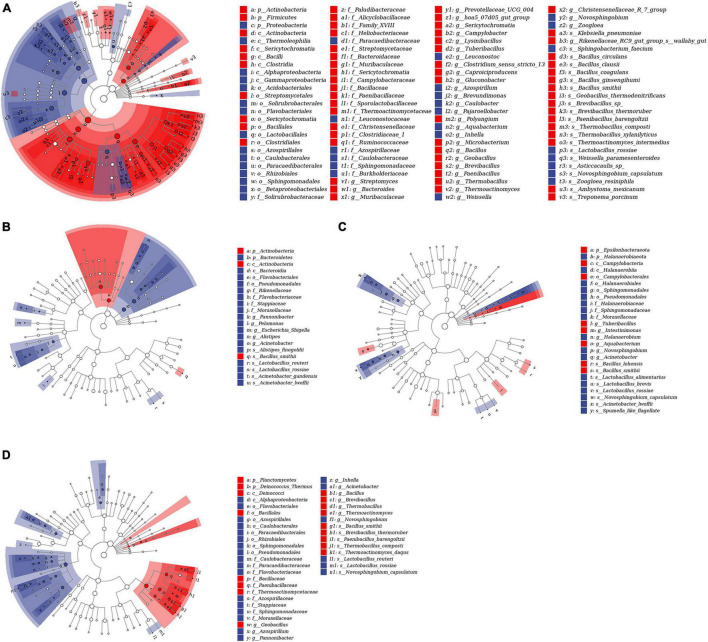
Linear discriminant analysis (LDA) effect size (LEfSe) analyses of bacterial communities in hybrid *Pennisetum* silage based on the threshold of LDA score > 3. **(A)** CK vs. MA; **(B)** CK vs. GL; **(C)** CK vs. CE; **(D)** CK vs. BS. CK, control group; MA, 1% fresh matter (FM) malic acid addition; GL, 1% FM glucose addition; CE, 100 U/g FM cellulase addition; BS, 10^6^ cfu/g *B. subtilis* FM addition.

To study the differential OTUs and potential biomarkers, we performed LEfSe analyses between CK and each additive treatment. As shown in Figure 3 and Supplementary Table 3, the differential OTUs between CK and MA were highest, and the addition of malic acid caused the upregulation of 81 differential OTUs and the downregulation of 48 differential OTUs (Figure 3A). Glucose caused the least number of differential OTUs, and the numbers of upregulated and downregulated OTUs were 3 and 18, respectively (Figure 3C). The number of differential OTUs caused by cellulase was 8 for upregulation and 17 for downregulation, and 17 upregulated and 23 downregulated OTUs were caused by *B. subtilis* (Figures 3B,D).

### Predicted Functions and Pathways of Bacterial Community in Hybrid *Pennisetum* Silage

**FIGURE 4:**
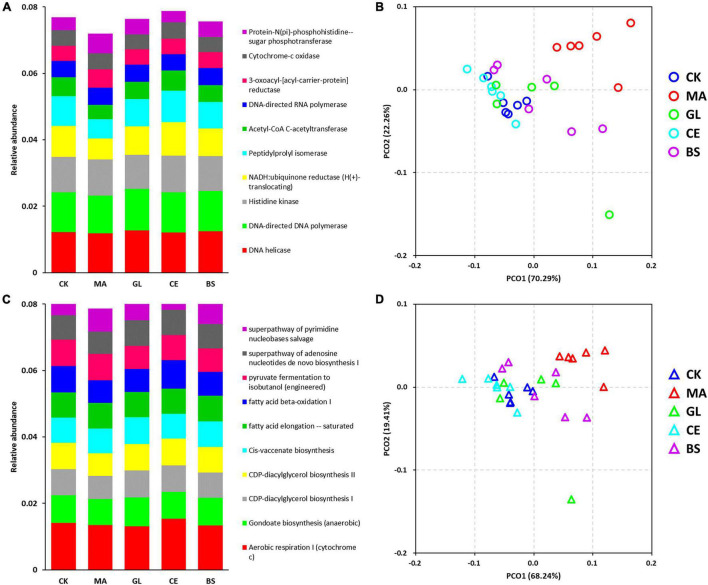
Effects of silage additives on the function of bacterial communities predicted by PICRUSt in hybrid *Pennisetum* silage. Panels **(A,C)** are the accumulation bar graphs of the top 10 functions and pathways; **(B,D)** are principal co-ordinates analysis (PCoA) scores plots on the function and pathway level. CK, control group; MA, 1% fresh matter (FM) malic acid addition; GL, 1% FM glucose addition; CE, 100 U/g FM cellulase addition; BS, 10^6^ cfu/g *B. subtilis* FM addition.

In this study, the PICRUSt was adopted for the functional prediction of bacterial communities (Figure 4 and Supplementary Table 4). The top five predicted functions were DNA helicase, DNA-directed DNA polymerase, histidine kinase, NADH:ubiquinone reductase (H^+^-translocating), and peptidylprolyl isomerase (Figure 4A). The top five pathways were aerobic respiration I (cytochrome c), gondoate biosynthesis (anaerobic), CDP–diacylglycerol biosynthesis I, CDP–diacylglycerol biosynthesis II, and *cis*-vaccenate biosynthesis (Figure 4C). The diversity of functions and pathways were also calculated, and the PCoA score plots of functions and pathways indicated that treated individuals were separated from four other treatments. The Adonis analyses further confirmed that malic acid played important roles in altering microbial functions (Table 4), but the effects caused by glucose, cellulase, and *B. subtilis* were unnoticeable.

**TABLE 4:** Adonis analyses of the components and functions of bacterial communities in silage hybrid *Pennisetum.*

Items	OTUs		Predicted functions		Predicted pathways	
	*R* ^2^	*P*	*R* ^2^	*P*	*R* ^2^	*P*
CK vs. MA	0.5924	0.010	0.7522	0.020	0.7195	0.017
CK vs. GL	0.1402	0.116	0.1413	0.237	0.1348	0.199
CK vs. CE	0.1646	0.039	0.2395	0.111	0.2382	0.085
CK vs. BS	0.2327	0.026	0.2260	0.141	0.2544	0.085

*CK, control group; MA, 1% FM malic acid addition; GL, 1% FM glucose addition; CE, 100 U/g FM cellulase addition; BS, 10^6^ cfu/g Bacillus subtilis FM addition. FM, fresh matter. The functions and pathways were predicted via PICRUSt.*

**FIGURE 5:**
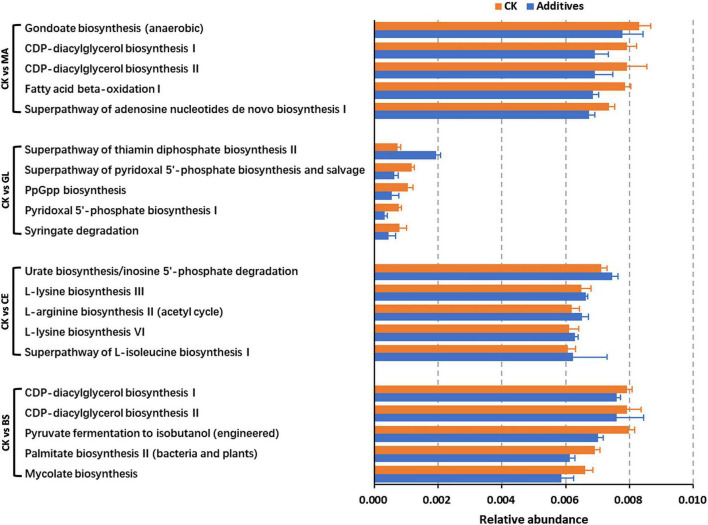
The top five pathways with statistical difference obtained by comparing CK and additive treatments. CK, control group; MA, 1% fresh matter (FM) malic acid addition; GL, 1% FM glucose addition; CE, 100 U/g FM cellulase addition; BS, 10^6^ cfu/g *B. subtilis* FM addition.

**FIGURE 6:**
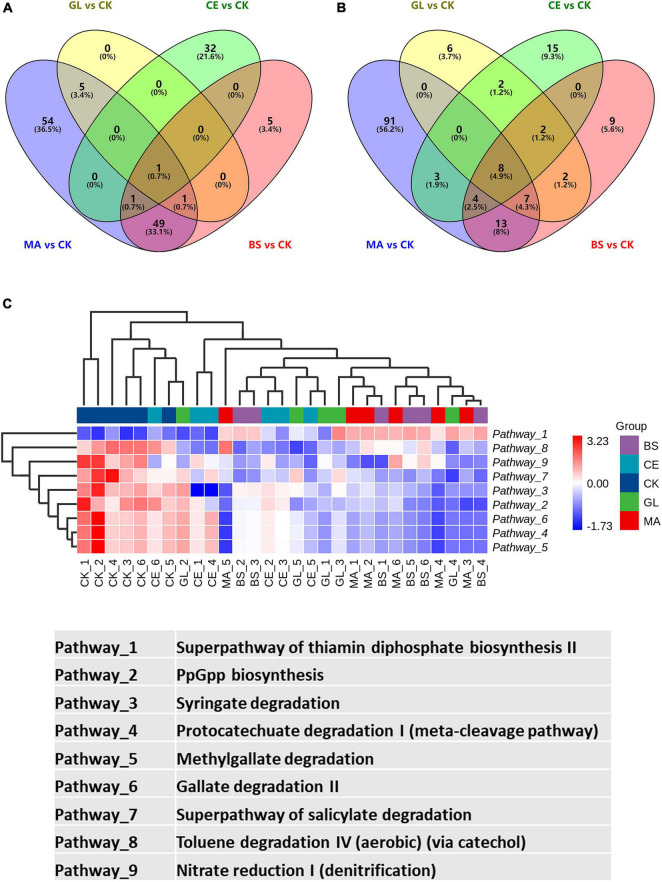
Differential pathways presented in CK vs. MA, CK vs. GL, CK vs. CE, and CK vs. BS. **(A)** Venn diagram of upregulated pathways; **(B)** venn diagram of downregulated pathways; **(C)** heatmap of the nine pathways affected by the four additives. CK, control group; MA, 1% fresh matter (FM) malic acid addition; GL, 1% FM glucose addition; CE, 100 U/g FM cellulase addition; BS, 10^6^ cfu/g *B. subtilis* FM addition.

The top five differential pathways between CK and four additive treatments are shown in Figure 5 and Supplementary Table 5. For malic acid and *B. subtilis*, the application of the two additives reduced the abundance of the top five differential pathways (*p* < 0.05), whereas the abundance of pathways were upregulated by cellulase (*p* < 0.05). Glucose upregulated the abundance of the superpathway of thiamin diphosphate biosynthesis II but downregulated the abundance of the superpathway of pyridoxal 5′-phosphate biosynthesis and salvage, PpGpp biosynthesis, pyridoxal 5′-phosphate biosynthesis I, and Syringate degradation (*p* < 0.05). In addition, we could find that the abundance of differential pathways in CK vs. GL were less than those in CK vs. MA, CK vs. CE, and CK vs. BS, implying that the effects of glucose on the dominant pathways were not evident. By analyzing all differential pathways, we found that nine pathways were simultaneously affected by all four additives (Figure 6 and Supplementary Table 6).

### Metabolic Profile in Hybrid *Pennisetum* Silage Affected by Silage Additives

The relative standard deviations (RSD) of the ion peak abundance in QC samples are displayed in Supplementary Figure 1A (positive ion mode) and Supplementary Figure 1B (negative ion mode). In this study, the number of peaks with RSD ≤ 30% in QC samples accounted for more than 80% of the total number of peaks, indicating that the instrumental analysis system was stable and reliable (Shen et al., 2016). The PCA score plots of 30 samples and 4 QC samples are shown in Supplementary Figure 2A (positive ion mode) and Supplementary Figure 2B (negative ion mode). Results showed that QC samples were closely clustered, suggesting that the repeatability of the experiment was good.

**FIGURE 7:**
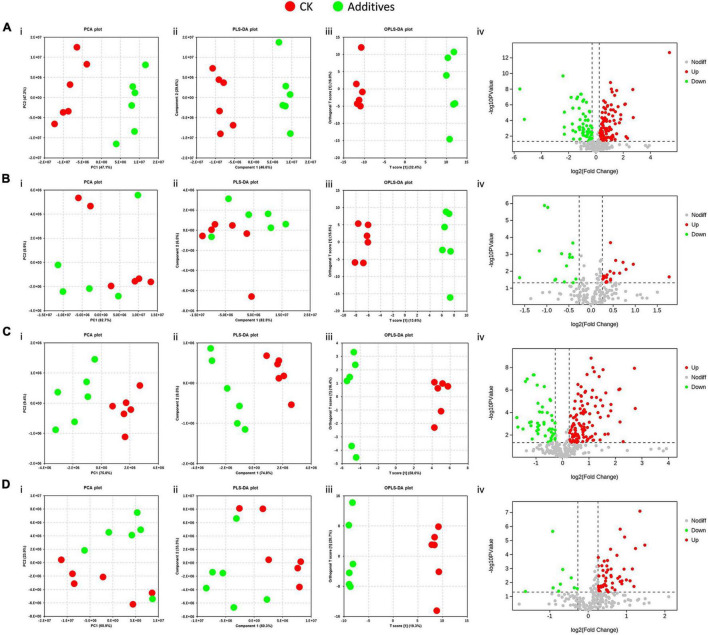
Effects of silage additives on the metabolic profile of bacterial communities predicted by PICRUSt in hybrid *Pennisetum* silage. **(A)** CK vs. MA; **(B)** CK vs. GL; **(C)** CK vs. CE; **(D)** CK vs. BS. **(i)** Principal component analysis (PCA) scores plots; **(ii)** projections to latent structures-discriminant analysis (PLS-DA) scores plots; **(iii)** orthogonal PLS-DA (OPLS-DA) scores plots; **(iv)** volcano plots. CK, control group; MA, 1% fresh matter (FM) malic acid addition; GL, 1% FM glucose addition; CE, 100 U/g FM cellulase addition; BS, 10^6^ cfu/g *B. subtilis* FM addition.

In this study, 422 metabolites, including 259 in the positive ion mode and 163 in the negative ion mode, were obtained. These metabolites could be divided into organic acids and derivatives, organic oxygen compounds, lipids and lipid-like molecules, organoheterocyclic compounds, benzenoids, and five other types (Supplementary Figure 3). Multivariate statistical analyses were conducted to distinguish the difference between CK and the four other treatments. PCA and PLS-DA score plots showed that the effects of malic acid and cellulase on the metabolic profile in HPS were evident, but the differences between CK and GL and between CK and BS were not significant (Figure 7). Subsequently, we performed OPLS-DA analyses, and the VIP values of each metabolite were calculated. After screening with the criterion of VIP > 1, FC > 1.2 or <0.833, and *p* < 0.05, differential metabolites were obtained (Supplementary Table 7). Malic acid upregulated 29 metabolites and downregulated 18 metabolites, and glucose upregulated 8 metabolites and downregulated 5 metabolites. The numbers of upregulated and downregulated metabolites caused by cellulase were 35 and 12, respectively. The numbers of upregulated and downregulated metabolites caused by *B. subtilis* were 16 and 2, respectively (Figure 7).

**FIGURE 8:**
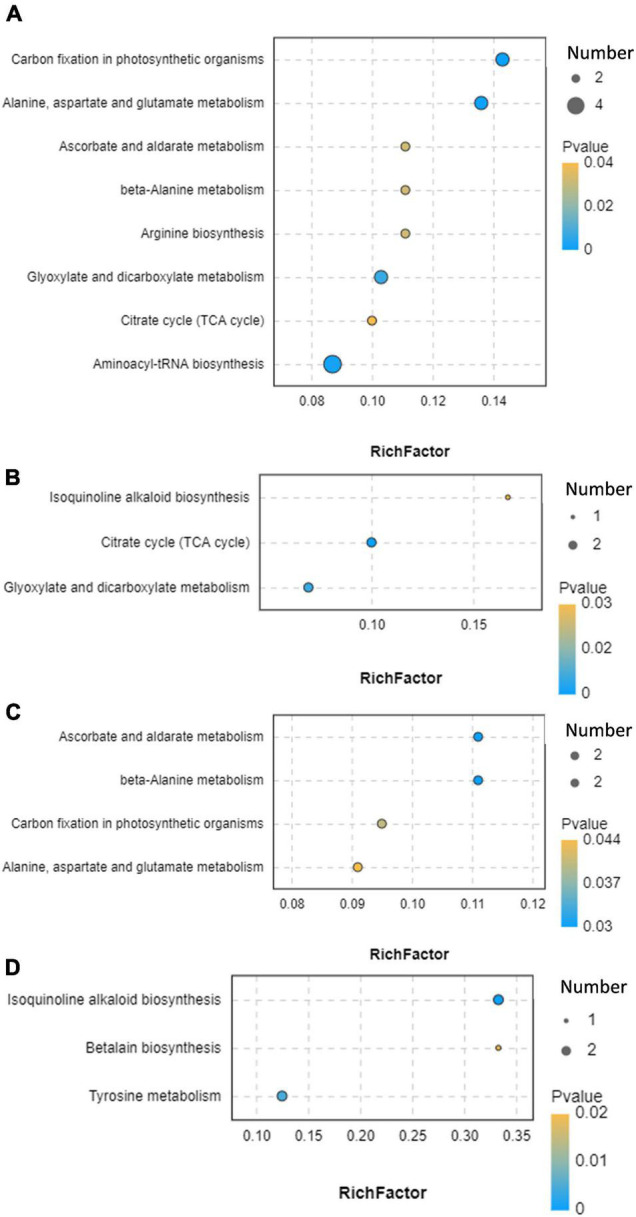
Functional annotation of differential metabolites. **(A)** CK vs. MA; **(B)** CK vs. GL; **(C)** CK vs. CE; **(D)** CK vs. BS. CK, control group; MA, 1% fresh matter (FM) malic acid addition; GL, 1% FM glucose addition; CE, 100 U/g FM cellulase addition; BS, 10^6^ cfu/g *B. subtilis* FM addition.

We further performed the functional annotation of differential metabolites *via* the KEGG database (Figure 8 and Supplementary Table 8). Results showed that differential metabolites were involved in carbon fixation in photosynthetic organisms; alanine, aspartate, and glutamate metabolism; aminoacyl-tRNA biosynthesis; glyoxylate and dicarboxylate metabolism; ascorbate and aldarate metabolism; beta-alanine metabolism; arginine biosynthesis; citrate cycle (TCA cycle); isoquinoline alkaloid biosynthesis; tyrosine metabolism; and betalain biosynthesis.

### Correlation Analyses Between Differential Operational Taxonomic Units and Metabolites

**FIGURE 9:**
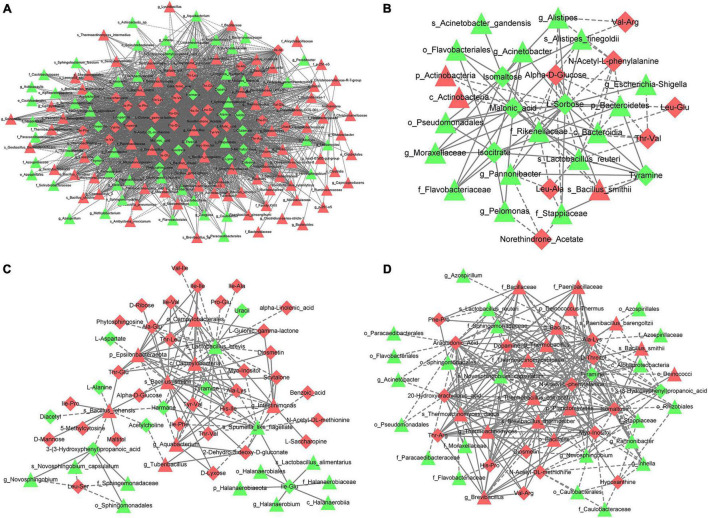
Correlation analysis between differential OTUs and metabolites. The differential OTUs are displayed as triangles and the differential metabolites are shown as diamonds. Red represents upregulation while green means downregulation. **(A)** CK vs. MA; **(B)** CK vs. GL; **(C)** CK vs. CE; **(D)** CK vs. BS. CK, control group; MA, 1% fresh matter (FM) malic acid addition; GL, 1% FM glucose addition; CE, 100 U/g FM cellulase addition; BS, 10^6^ cfu/g *B. subtilis* FM addition.

In the comparison between CK and MA, g_*Thermobacillus* was positively correlated with alanine–glucose (Cor = 0.9793, *p* = 2.92E−08) but negatively correlated with L-alanine (Cor = −0.9510, *p* = 2.05E−06) and D-threitol (Cor = −0.9419, *p* = 4.71E−06; Figure 9A). For CK vs. GL, s_*Alistipes finegoldii* was positively correlated with isomaltose (Cor = 0.8647, *p* = 5.92E−04), and f_*Rikenellaceae* was negatively correlated with alpha-D-glucose (Cor = −0.8351, *p* = 1.38E−03) and *N*-acetyl-L-phenylalanine (Cor = −0.7928, *p* = 3.60E−03; Figure 9B). In the comparison between CK and CE, s_*Bacillus smithii* was positively correlated with threonine–valine (Cor = 0.9085, *p* = 4.32E−05) and 5-methylcytosine (Cor = 0.8798, *p* = 1.61E−04), and s_*Lactobacillus brevis* was negatively correlated with isoleucine–isoleucine (Cor = −0.7959, *p* = 1.96E−03) and proline–glucose (Cor = −0.7741, *p* = 3.12E−03, Figure 9C). For CK vs. BS, s_*Brevibacillus thermoruber* was positively correlated with histidine–proline (Cor = 0.8935, *p* = 8.98E−05) and isomaltose (Cor = 0.8850, *p* = 1.30E−04), and tyramine was negatively correlated with g_*Thermoactinomyces* and f_*Thermoactinomycetaceae* (Cor = −0.9297, *p* = 1.20E−05; Cor = −0.9232, *p* = 1.85E−05; Figure 9D). Other correlations are displayed in Supplementary Table 9.

## Discussion

### Characteristics of Fresh Hybrid *Pennisetum*

In this study, the DM content of the hybrid *Pennisetum* was 336.30 g/kg FM, which met the ideal DM content of 30–35% (Guyader et al., 2018). NDF, ADF, and CP contents were all higher than that reported by Shah et al. (2020a). The variation might be attributed to the geographical and climatic conditions for plant growth and the growth stage of a plant. The WSC content of hybrid *Pennisetum* was 26.84 g/kg DM, which was insufficient to meet the minimum requirement of 60–70 g/kg DM for well-preserved silage (Wang et al., 2021). As a result, the moisture content of hybrid *Pennisetum* was suitable for silage, but the insufficient WSC might prevent continuous fermentation.

### Fermentation Parameters and Chemical Compositions of Hybrid *Pennisetum* Silage

Dry matter losses are caused by the metabolic activities of aerobic microorganisms, and digestible carbohydrates are an important fraction for consumption (Yuan et al., 2017). The addition of glucose might alleviate the consumption of carbohydrates to some extent. The pH values of HPS in all five treatments were less than 4.2 and could be considered as well fermented (He et al., 2020). Malic acid reduced the pH due to its acidity, and the decreased pH in CE might be attributed to increased AA. Cellulase can hydrolyze structural carbohydrates and provide a fermentation substrate for LAB, thereby accumulating organic acids and reducing pH (Li F. et al., 2020). The changes in the contents of LA and AA in CK and MA might be because of different fermentation types. WSC was consumed and decomposed into organic acids under the activities of microorganisms during the first 30 days of silage, and the fermentation in GL, CE, and BS might be complete (Chen et al., 2020). Similar to WSC, starch is a type of rapidly degradable carbohydrate and can provide a substrate for fermentation (Feng et al., 2020). The CP content in CE was highest, which might be due to the carbon loss during fermentation (He et al., 2018). In addition, silage additives had remarkable effects on the nitrogen fractions of HPS. AN is produced by clostridial fermentation, and one of the criteria for good silage is AN < 100 g/kg TN (Mu et al., 2020). NPN is an important product of proteolysis, and the utilization rate of NPN in ruminants is lower compared with that of TP due to rapid degradation (Dentinho et al., 2019; Wang et al., 2021). The present study suggested that the addition of malic acid and cellulase had positive effects on inhibiting proteolysis, and low pH was speculated to inhibit the activities of proteases (He et al., 2020). NDIN and ADIN belong to bonding proteins, which bond plants with cell wall. NDIN is degraded and utilized in the rumen slowly, whereas ADIN cannot be utilized (Licitra et al., 1996). As a result, the four additives used in this study had positive effects on the nutrients of HPS, and this finding was manifested in increased available protein and reduced structural carbohydrates.

### Bacterial Diversity in Hybrid *Pennisetum* Silage Affected by Silage Additives

Given the important influence of microorganisms on silage fermentation, bacterial communities should be monitored. The α-diversity refers to the richness and diversity of species in a particular habitat or ecosystem and can indicate the degree of species isolation in the habitat. According to Bai et al. (2020), when dominant bacteria are abundant, the diversity of microbial community is low. In this study, the dominant bacteria at the phylum (*Proteobacteria*) and genus (*Aquabacterium*) levels in CE were highest among the five treatments, whereas the dominant bacteria in MA were lowest. These findings might be the reason why the α-diversity indices in CK were lower and higher than those in MA and CE, respectively. The β-diversity can reflect the differences between and within groups. Results indicated that malic acid, cellulase, and *B. subtilis* had significant effects on the bacterial communities of the HPS. However, the effects of glucose seemed inconspicuous.

### Bacterial Abundance in Hybrid *Pennisetum* Silage Affected by Silage Additives

Bacterial communities were altered by silage additives, whereas *Firmicutes* and *Proteobacteria* were always the dominant phyla. Results were consistent with those reported by Wang et al. (2021); Zhang et al. (2021), and Zi et al. (2021). Almost all LAB belong to *Firmicutes*, which are involved in the degradation of biomacromolecules and secretes various lipases, cellulases, and proteases (Yuan et al., 2020). The upregulation of Firmicutes in MA might lead to increased LA. A wide variety of pathogenic Gram-negative bacteria, including *Escherichia*, *Helicobacter*, *Legionellales*, *Salmonella*, *Vibrio*, and *Yersinia*, belong to *Proteobacteria*. Malic acid may inhibit pathogenic bacteria (Du et al., 2021). Moreover, *Actinobacteria* and *Deinococcus–Thermus* were upregulated by malic acid and cellulase, respectively. However, research on the two phyla in silage remains limited, and mechanisms need further research.

At the genus level, *Aquabacterium* was the dominant bacteria and accounted for the vast majority of *Proteobacteria*. According to previous reports, *Aquabacterium* is a facultatively anaerobic, non-spore-forming, and rod-shaped bacteria detected in soil and fresh water, and most species cannot metabolize carbohydrates (Chen et al., 2012; Hirose et al., 2020). Thus far, only Xu et al. (2020) reported *Aquabacterium* in silage. Therefore, the function of the genus remains unknown. *Bacillus*, a type of facultatively anaerobic and Gram-positive bacteria, can secrete bacteriocin and inhibit the activities of undesirable bacteria. In addition, some species of *Bacillus* can produce LA (Li M. et al., 2020). *Weissella*, a type of heterofermentative LAB, converts 1 mol glucose to 1 mol AA, 1 mol LA, and 1 mol CO_2_ (Wang M. et al., 2019). The upregulation of *Bacillus* and downregulation of *Weissella* may lead to increased LA and decreased AA in MA. Some species of *Brevibacillus* are reported to produce antibacterial agents (Miljkovic et al., 2019). The antimicrobial activities of malic acid might be attributed to the upregulation of *Bacillus*, *Brevibacillus*, and other similar bacteria.

The LEfSe analysis is a tool for the interpretation and discovery of high-dimensional data biomarkers, highlights biological correlation and statistical significance, and can explore potential biomarkers that are different among groups. In this study, only two differential bacteria were affected by all four additives. *B. smithii* was upregulated, whereas *Lactobacillus rossiae* was downregulated, indicating that the four additives had different regulating mechanisms on silage quality. *B. smithii* can convert cellulose into LA *via* simultaneous saccharification and fermentation processes (Chacón et al., 2021). Although research about *B. smithii* on silage remains limited, its characteristics of simultaneous saccharification and fermentation allow increased fermentation efficiency. *B. smithii* may be developed as a new silage or food additive, but the safety of produced silage or food should be assessed. *L. rossiae*, a type of LAB, converts arginine into toxic putrescine *via* the ornithine decarboxylase pathway (Del Rio et al., 2018). *L. rossiae* may be a biomarker of silage safety and quality, but no direct evidence shows that *L. rossiae* is related to silage spoilage and accumulation of toxins. Notably, *Moraxellaceae* is inhibited by glucose, cellulase, and *B. subtilis* and previously reported as a pathogenic microorganism (Narciso-da-Rocha et al., 2018). *Lysinibacillus* and *Paenibacillus* were upregulated by malic acid. These bacilli are reported to be probiotics and considered as potential fermentation additives (Liñan-Vidriales et al., 2021; Soni and Keharia, 2021). *Methylobacterium* is a strictly aerobic bacterium and involved in environmental carbon cycle and decomposition of compounds in plants (Wang et al., 2021). Ali et al. (2020) reported that *Methylobacterium* is downregulated from the pre-ensiled to the post-ensiled period, and the reduction of the genus implies that malic acid may create good conditions for fermentation. *Leuconostoc* is a lactate-producing bacteria (Wang et al., 2021). However, with increasing LA in MA, the abundance of the genus declined. This finding was speculated to be caused by competition between LAB, and the growth of dominant LAB might inhibit the metabolic activities of LAB with low abundance. The addition of glucose reduced the relative abundance of some pathogenic bacteria, including *Escherichia–Shigella* and *Acinetobacter* (Liu et al., 2020; De Almeida et al., 2021). Similarly, *Acinetobacter* was downregulated by *B. subtilis*. *Pseudomonadales* was downregulated by cellulase. According to a previous report, some members of the order are spoilage microorganisms with lipolytic and proteolytic enzymatic activities (Porcellato et al., 2018). This finding might explain why CE had the highest CP and TP contents among the five treatments. In summary, the results of LEfSe analyses implied that bacterial communities might be optimized by four additives. However, the mechanisms required in-depth research because of the complex competition and cooperation in microorganisms.

### Predicted Functions and Pathways of Bacterial Community in Hybrid *Pennisetum* Silage

The function of bacterial communities was determined by their composition to a high extent. In this study, malic acid had significant effects on the functions of bacterial communities, as evidenced by the addition of malic acid caused the most differential bacteria compared with the addition of glucose, cellulase, and *B. subtilis*. This finding might be because the malic acid additive led to abundant variations in some functional bacteria (Wang et al., 2021). Malic acid has antioxidant and antibacterial functions and is widely used in food, pharmaceutical, healthcare, cosmetics, and other industries. Moreover, malic acid, which can be used as a carbohydrate source, provides energy for LAB and accelerates LAB growth (Li M. et al., 2020). Notably, the relative abundance of differential pathways caused by glucose was lower compared with those of pathways caused by malic acid, cellulase, and *B. subtilis*. Thus, glucose had little effect on dominant pathways. Nine pathways, including 1 upregulated (i.e., superpathway of thiamin diphosphate biosynthesis II) and 8 downregulated (i.e., PpGpp biosynthesis, syringate degradation, protocatechuate degradation I [meta-cleavage pathway], methyl gallate degradation, gallate degradation II, superpathway of salicylate degradation, toluene degradation IV [aerobic, *via* catechol], and nitrate reduction I [denitrification]) pathways, were affected by all four additives. We hypothesized that the nine pathways were the core pathways affecting silage quality in the hybrid *Pennisetum*.

### Metabolic Profile in Hybrid *Pennisetum* Silage Affected by Silage Additives

Ensiling is a complex biological fermentation process that involves a wide variety of microorganisms, thereby leading to many kinds of metabolites. Conventionally, VFAs and LA are adopted for evaluating silage quality, whereas other metabolites are also of concern. Therefore, metabolomics technology can provide a complete understanding of metabolites in the microenvironment. The addition of glucose caused 13 differential metabolites, which was the least among the four additives. CK and GL could not be separated in PCA and PLS-DA score plots, which might be because glucose had less effect on the microflora and caused few differential metabolites. No metabolite was simultaneously upregulated or downregulated by all four additives. This finding was consistent with the fact that the four additives shared few differential bacteria and further suggested that the mechanisms of the four additives altering silage quality varied. In addition, the functional annotation based on KEGG database showed that the differential metabolites were involved in the metabolism and biosynthesis of small molecules, TCA cycle, and carbon fixation in photosynthetic organisms. This was beneficial to the comprehension of metabolites in silage and contributed to the identification of beneficial metabolites (Guan et al., 2020).

A total of 26 metabolites were obtained in MA vs. CK and CE vs. CK, indicating that malic acid and cellulase had the most similar effects on the metabolic profiles of HPS. Besides, malic acid and cellulase affected four pathways, including carbon fixation in photosynthetic organisms; alanine, aspartate, and glutamate metabolism; ascorbate and aldarate metabolism; and beta-alanine metabolism. Considering that malic acid and cellulase reduced the ratio of AN and NPN in HPS, we speculated that the four pathways might play some unknown functions. Among the four pathways, five differential metabolites, including L-alanine, L-aspartate, L-gulono-1,4-lactone, myo-inositol, and uracil, were obtained. In this study, L-aspartate was involved in five pathways and negatively related to *B. smithii*. Coincidentally, this species was negatively related to L-alanine, which played important roles in three pathways. This result confirmed the important role of *B. smithii* in silage. In the research of metabolome on silage, amino acids as metabolites are reported, whereas the functions of amino acids are still unknown (Guo et al., 2018; Xu et al., 2019). Besides, we found that malic acid and cellulase could upregulate the expression of several dipeptides, like alanine–glucose, threonine–glucose, histidine–isoleucine, and proline–glucose. Some dipeptides are reported to exert anti-inflammatory functions and considered as flavor substances (Gallego et al., 2021; Molinari et al., 2021). Thus, silage supplemented with malic acid and cellulase might provide improved palatability and animal health. *Tuberibacillus*, *B. smithii*, and *Bacillus lehensis* might play positive roles in the production of dipeptides. In addition, as an important part of the TCA cycle, malic acid can directly regulate the metabolism of carbohydrates and proteins through this biochemical reaction (Li M. et al., 2020). Two differential metabolites (i.e., malic acid and isocitrate) caused by glucose are involved in the TCA cycle and glyoxylate and dicarboxylate metabolism, which are reported to be related with carbohydrate metabolism (Ruan et al., 2021). According to the correlation analysis, bacteria associated with malic and isocitrate included *Pseudomonadales*, *Acinetobacter*, *Pelomonas*, and *Lactobacillus reuteri*, suggesting that these microorganisms might be potential targets for regulating carbohydrate metabolism during silage. For other metabolites, some special sugars are considered as biomarkers of poor fermentation. According to Li M. et al. (2021), the downregulation of isomaltose and L-sorbose may indicate high-quality ensiling. *B. subtilis* caused 18 differential metabolites. Among these metabolites, diosmetin is a flavonoid and has antioxidant, antibacterial, and anti-inflammatory properties (Lee et al., 2020). In the present study, diosmetin was positively related to *Thermoactinomyces daqus* and *Brevibacillus thermoruber*, suggesting that these two species might be involved in the production of diosmetin and other bioactive compounds. Several studies proved that different additives in silage can cause changes in bioactive metabolites, such as polyphenols, flavonoids, and terpenoids (Guan et al., 2020; Hu et al., 2020; Xu et al., 2021). However, these active substances are rarely used directly as silage additives. The effects of active substances on silage should be studied. Arachidonic acid (*cis*-5,8,11,14-eicosapentaenoic acid) is a type of ω-6 polyunsaturated fatty acid with anti-inflammatory functions (Trostchansky et al., 2021). Arachidonic acid was positively related to *Bacillus*, and the upregulation might be attributed to the inoculation of *B. subtilis*. Notably, correlation analyses were performed in accordance with statistical and correlation parameters. Therefore, results could not be regarded as causation (Xu et al., 2019). The present study provided potential biomarkers for improving the silage quality, and the development and application of amino acids, organic acid, small peptides, phenols, and other bioactive compounds will be the research focus of silage additives (Xu et al., 2019).

## Conclusion

The application of malic acid, glucose, cellulase, and *B. subtilis* promoted the fermentation quality and nutrient composition by altering the bacterial communities and metabolic profiles of HPS. In this study, the dominant phyla were *Firmicutes* and *Proteobacteria* and that the dominant genera were *Aquabacterium* and *Bacillus*. Malic acid, glucose, cellulase, and *B. subtilis* caused 129, 21, 25, and 40 differential bacteria, respectively, and 47, 13, 47, and 18 differential metabolites, respectively. Thereinto, *B. smithii* was upregulated by all the four additives and had potential to be used as a silage inoculant. These differential metabolites included amino acids, organic acids, sugars, small peptides, and phenols and were involved in various pathways, such as the TCA cycle; carbon fixation in photosynthetic organisms; alanine, aspartate, and glutamate metabolism; and glyoxylate and dicarboxylate metabolism. Some metabolites exerted antioxidant, anti-inflammatory, and antibacterial functions and had potential to be silage additives. In summary, the present study provided new suggestions on screening biomarkers for modulating silage quality.

## Data Availability Statement

Raw sequencing data sets are available in the NCBI Sequence Read Archive (SRA) under the BioProject accession PRJNA760648.

## Author Contributions

HT, YG, and MD conceived and designed the study. HT, YZ, MD, and TL performed the experiments. HT and YZ organized the database and performed the statistical analysis. HT, YZ, and MD wrote the article. MD and TL visualized the results. YG, MD, and BS revised the article. All authors read and approved the article.

## Conflict of Interest

The authors declare that the research was conducted in the absence of any commercial or financial relationships that could be construed as a potential conflict of interest.

## Publisher’s Note

All claims expressed in this article are solely those of the authors and do not necessarily represent those of their affiliated organizations, or those of the publisher, the editors and the reviewers. Any product that may be evaluated in this article, or claim that may be made by its manufacturer, is not guaranteed or endorsed by the publisher.

## Footnotes

1
http://huttenhower.sph.harvard.edu/galaxy/

## References

[B1] Ali N. Wang S. Zhao J. Dong Z. Li J. Nazar M. (2020). Microbial diversity and fermentation profile of red clover silage inoculated with reconstituted indigenous and exogenous epiphytic microbiota. *Bioresource Technol.* 314:123606. 10.1016/j.biortech.2020.123606 32629380

[B2] AOAC (2002). *Official Methods of Analysis*, 17th Edn. Gaithersburg, VA: Association of Official Analytical Chemists.

[B3] Bai J. Xu D. Xie D. Wang M. Li Z. Guo X. (2020). Effects of antibacterial peptide-producing *Bacillus subtilis* and *Lactobacillus buchneri* on fermentation, aerobic stability, and microbial community of alfalfa silage. *Bioresour. Technol.* 315:123881. 10.1016/j.biortech.2020.123881 32731157

[B4] Bakhshy E. Zarinkamar F. Nazari M. (2020). Structural and quantitative changes of starch in seed of Trigonella persica during germination. *Int. J. Biol. Macromol.* 164 1284–1293. 10.1016/j.ijbiomac.2020.07.262 32755696

[B5] Benton H. P. Ivanisevic J. Mahieu N. G. Kurczy M. E. Johnson C. H. Franco L. (2015). Autonomous metabolomics for rapid metabolite identification in global profiling. *Anal. Chem.* 87 884–891. 10.1021/ac5025649 25496351 PMC4303330

[B6] Cai C. Wang L. Wang G. Hao J. Bai X. Wang Z. (2020). Effects of dry explosion pretreatment on physicochemical and fuel properties of hybrid pennisetum (*Pennisetum americanum* × *P. purpureum*). *Bioresour. Technol.* 297:122508. 10.1016/j.biortech.2019.122508 31816573

[B7] Carvalho-Estrada P. D. Fernandes J. da Silva É. B. Tizioto P. Paziani S. D. Duarte A. P. (2020). Effects of hybrid, kernel maturity, and storage period on the bacterial community in high-moisture and rehydrated corn grain silages. *Syst. Appl. Microbiol.* 43:126131. 10.1016/j.syapm.2020.126131 32866836

[B8] Chacón M. G. Ibenegbu C. Leak D. J. (2021). Simultaneous saccharification and lactic acid fermentation of the cellulosic fraction of municipal solid waste using *Bacillus smithii*. *Biotechnol. Lett.* 43 667–675. 10.1007/s10529-020-03049-y 33219874 PMC7873104

[B9] Chen H. Jiang W. (2014). Application of high-throughput sequencing in understanding human oral microbiome related with health and disease. *Front. Microbiol.* 5:508. 10.3389/fmicb.2014.00508 25352835 PMC4195358

[B10] Chen L. Qu H. Bai S. Yan L. You M. Gou W. (2020). Effect of wet sea buckthorn pomace utilized as an additive on silage fermentation profile and bacterial community composition of alfalfa. *Bioresour. Technol.* 314:123773. 10.1016/j.biortech.2020.123773 32645569

[B11] Chen W.-M. Cho N.-T. Yang S.-H. Arun A. B. Young C.-C. Sheu S.-Y. (2012). *Aquabacterium limnoticum* sp. nov., isolated from a freshwater spring. *Int. J. Syst. Evol. Microbiol.* 62 698–704. 10.1099/ijs.0.030635-0 21551326

[B12] De Almeida A. G. G. Furlan J. P. R. Stehling E. G. De Martinis E. C. P. (2021). Comparative phylo-pangenomics reveals generalist lifestyles in representative *Acinetobacter* species and proposes candidate gene markers for species identification. *Gene* 791:145707. 10.1016/j.gene.2021.145707 33979679

[B13] De Filippis F. Parente E. Zotta T. Ercolini D. (2018). A comparison of bioinformatic approaches for 16S rRNA gene profiling of food bacterial microbiota. *Int. J. Food Microbiol.* 265 9–17. 10.1016/j.ijfoodmicro.2017.10.028 29107843

[B14] Del Rio B. Alvarez-Sieiro P. Redruello B. Martin M. C. Fernandez M. Ladero V. (2018). Lactobacillus rossiae strain isolated from sourdough produces putrescine from arginine. *Sci. Rep.* 8:3989. 10.1038/s41598-018-22309-6 29507315 PMC5838238

[B15] Dentinho M. T. P. Paulos K. Portugal P. V. Moreira O. C. Santos-Silva J. Bessa R. J. B. (2019). Proteolysis and *in situ* ruminal degradation of lucerne ensiled with *Cistus ladanifer* tannins. *Grass Forage Sci.* 74 78–85. 10.1111/gfs.12394

[B16] Du Z. Sun L. Chen C. Lin J. Yang F. Cai Y. (2021). Exploring microbial community structure and metabolic gene clusters during silage fermentation of paper mulberry, a high-protein woody plant. *Anim. Feed Sci. Technol.* 275:114766. 10.1016/j.anifeedsci.2020.114766

[B17] Feng X. Sun B. Yu P. (2020). Using vibrational molecular spectroscopy to detect moist heating induced carbohydrates structure changes in cool-climate adapted barley grain. *J. Cereal Sci.* 95:103007. 10.1016/j.jcs.2020.103007 PMC7274425 32502162

[B18] Gallego M. Toldrá F. Mora L. (2021). Quantification and in silico analysis of taste dipeptides generated during dry-cured ham processing. *Food Chem.* 370 130977. 10.1016/j.foodchem.2021.130977 34509941

[B19] Gill S. R. Pop M. DeBoy R. T. Eckburg P. B. Turnbaugh P. J. Samuel B. S. (2006). Metagenomic analysis of the human distal gut microbiome. *Science* 312:1355–1359. 10.1126/science.1124234 16741115 PMC3027896

[B20] Guan H. Shuai Y. Ran Q. Yan Y. Wang X. Li D. (2020). The microbiome and metabolome of Napier grass silages prepared with screened lactic acid bacteria during ensiling and aerobic exposure. *Anim. Feed Sci. Technol.* 269:114673. 10.1016/j.anifeedsci.2020.114673

[B21] Guo X. S. Ke W. C. Ding W. R. Ding L. M. Xu D. M. Wang W. W. (2018). Profiling of metabolome and bacterial community dynamics in ensiled *Medicago sativa* inoculated without or with *Lactobacillus plantarum* or *Lactobacillus buchneri*. *Sci. Rep.* 8:357. 10.1038/s41598-017-18348-0 29321642 PMC5762819

[B22] Guyader J. Baron V. S. Beauchemin K. A. (2018). Corn forage yield and quality for silage in short growing season areas of the canadian prairies. *Agronomy* 8:164. 10.3390/agronomy8090164

[B23] Hao Y. Wang X. Yuan S. Wang Y. Liao X. Zhong M. (2021). Flammulina velutipes polysaccharide improves C57BL/6 mice gut health through regulation of intestine microbial metabolic activity. *Int. J. Biol. Macromol.* 167 1308–1318. 10.1016/j.ijbiomac.2020.11.085 33202270

[B24] He L. Chen N. Lv H. Wang C. Zhou W. Chen X. (2020). Gallic acid influencing fermentation quality, nitrogen distribution and bacterial community of high-moisture mulberry leaves and stylo silage. *Bioresour. Technol.* 295:122255. 10.1016/j.biortech.2019.122255 31639626

[B25] He L. Zhou W. Wang Y. Wang C. Chen X. Zhang Q. (2018). Effect of applying lactic acid bacteria and cellulase on the fermentation quality, nutritive value, tannins profile and *in vitro* digestibility of *Neolamarckia cadamba* leaves silage. *J. Anim. Physiol. Anim. Nutr.* 102 1429–1436. 10.1111/jpn.12965 30062737

[B26] Hirose S. Tank M. Hara E. Tamaki H. Mori K. Takaichi S. (2020). *Aquabacterium pictum* sp. nov., the first aerobic bacteriochlorophyll a-containing fresh water bacterium in the genus *Aquabacterium* of the class *Betaproteobacteria*. *Int. J. Syst. Evol. Microbiol.* 70 596–603. 10.1099/ijsem.0.003798 31622237

[B27] Hu Z. Niu H. Tong Q. Chang J. Yu J. Li S. (2020). The microbiota dynamics of alfalfa silage during ensiling and after air exposure, and the metabolomics after air exposure are affected by Lactobacillus casei and cellulase addition. *Front. Microbiol.* 11:519121. 10.3389/fmicb.2020.519121 33329411 PMC7732661

[B28] Langille M. G. I. Zaneveld J. Caporaso J. G. McDonald D. Knights D. Reyes J. A. (2013). Predictive functional profiling of microbial communities using 16S rRNA marker gene sequences. *Nat. Biotechnol.* 31 814–821. 10.1038/nbt.2676 23975157 PMC3819121

[B29] Lee D. Park J. Choi J. Jang H. Seol J. (2020). Anti-inflammatory effects of natural flavonoid diosmetin in IL-4 and LPS-induced macrophage activation and atopic dermatitis model. *Int. Immunopharmacol.* 89:107046. 10.1016/j.intimp.2020.107046 33045572 PMC7545212

[B30] Li F. Ke W. Ding Z. Bai J. Zhang Y. Xu D. (2020). Pretreatment of *Pennisetum sinese* silages with ferulic acid esterase-producing lactic acid bacteria and cellulase at two dry matter contents: fermentation characteristics, carbohydrates composition and enzymatic saccharification. *Bioresour. Technol.* 295:122261. 10.1016/j.biortech.2019.122261 31645008

[B31] Li M. Zhang L. Zhang Q. Zi X. Lv R. Tang J. (2020). Impacts of citric acid and malic acid on fermentation quality and bacterial community of cassava foliage silage. *Front. Microbiol.* 11:595622. 10.3389/fmicb.2020.595622 33424799 PMC7793898

[B32] Li R. Sun Z. Zhao Y. Li L. Yang C. Cen J. (2021). Application of UHPLC-Q-TOF-MS/MS metabolomics approach to investigate the taste and nutrition changes in tilapia fillets treated with different thermal processing methods. *Food Chem.* 356:129737. 10.1016/j.foodchem.2021.129737 33836358

[B33] Li M. Lv R. Zhang L. Zi X. Zhou H. Tang J. (2021). Melatonin is a promising silage additive: evidence from microbiota and metabolites. *Front. Microbiol.* 12:670764. 10.3389/fmicb.2021.670764 34122385 PMC8187806

[B34] Licitra G. Hernandez T. M. Van Soest P. J. (1996). Standardization of procedures for nitrogen fractionation of ruminant feeds. *Anim. Feed Sci. Technol.* 57 347–358. 10.1016/0377-8401(95)00837-3

[B35] Liñan-Vidriales M. A. Peña-Rodríguez A. Tovar-Ramírez D. Elizondo-González R. Barajas-Sandoval D. R. Ponce-Gracía E. I. (2021). Effect of rice bran fermented with *Bacillus* and *Lysinibacillus* species on dynamic microbial activity of Pacific white shrimp (*Penaeus vannamei*). *Aquaculture* 531:735958. 10.1016/j.aquaculture.2020.735958

[B36] Liu Q. X. Zhou Y. Li X. M. Dan D. Xing S. Feng J. H. (2020). Ammonia induce lung tissue injury in broilers by activating NLRP3 inflammasome *via Escherichia*/*Shigella*. *Poult. Sci.* 99 3402–3410. 10.1016/j.psj.2020.03.019 32616234 PMC7597683

[B37] Malik I. Batra T. Das S. Kumar V. (2020). Light at night affects gut microbial community and negatively impacts host physiology in diurnal animals: evidence from captive zebra finches. *Microbiol. Res.* 241:126597. 10.1016/j.micres.2020.126597 32979783

[B38] McAllister T. A. Dunière L. Drouin P. Xu S. Wang Y. Munns K. (2018). Silage review: using molecular approaches to define the microbial ecology of silage. *J. Dairy Sci.* 101 4060–4074. 10.3168/jds.2017-13704 29685277

[B39] Miljkovic M. Jovanovic S. O’Connor P. M. Mirkovic N. Jovcic B. Filipic B. (2019). *Brevibacillus laterosporus* strains BGSP7, BGSP9 and BGSP11 isolated from silage produce broad spectrum multi-antimicrobials. *PLoS One* 14:e0216773. 10.1371/journal.pone.0216773 31075157 PMC6510442

[B40] Molinari G. S. Wojno M. McCracken V. J. Kwasek K. (2021). The use of dipeptide supplementation as a means of mitigating the negative effects of dietary soybean meal on Zebrafish Danio rerio. *Comp. Biochem. Physiol. A Mol. Integr. Physiol.* 257:110958. 10.1016/j.cbpa.2021.110958 33865992

[B41] Mu L. Xie Z. Hu L. Chen G. Zhang Z. (2020). Cellulase interacts with *Lactobacillus plantarum* to affect chemical composition, bacterial communities, and aerobic stability in mixed silage of high-moisture amaranth and rice straw. *Bioresour. Technol.* 315:123772. 10.1016/j.biortech.2020.123772 32653750

[B42] Muck R. E. Nadeau E. M. G. McAllister T. A. Contreras-Govea F. E. Santos M. C. Kung L. Jr. (2018). Silage review: recent advances and future uses of silage additives. *J. Dairy Sci.* 101 3980–4000. 10.3168/jds.2017-13839 29685273

[B43] Narciso-da-Rocha C. Rocha J. Vaz-Moreira I. Lira F. Tamames J. Henriques I. (2018). Bacterial lineages putatively associated with the dissemination of antibiotic resistance genes in a full-scale urban wastewater treatment plant. *Environ. Int.* 118 179–188. 10.1016/j.envint.2018.05.040 29883764

[B44] Porcellato D. Aspholm M. Skeie S. B. Monshaugen M. Brendehaug J. Mellegård H. (2018). Microbial diversity of consumption milk during processing and storage. *Int. J. Food Microbiol.* 266 21–30. 10.1016/j.ijfoodmicro.2017.11.004 29161642

[B45] Ruan X. Deng X. Tan M. Wang Y. Hu J. Sun Y. (2021). Effect of resveratrol on the biofilm formation and physiological properties of avian pathogenic *Escherichia coli*. *J. Proteomics* 249 104357. 10.1016/j.jprot.2021.104357 34450330

[B46] Rumsey T. S. Noller C. H. Rhykerd C. L. Burns J. C. (1967). Measurement of certain metabolic organic acids in forage, silage, and ruminal fluid by gas-liquid chromatography. *J. Dairy Sci.* 50 214–219. 10.3168/jds.s0022-0302(67)87390-9

[B47] Shah A. A. Liu Z. Qian C. Wu J. Zhong X. Kalsoom U. (2020a). Effect of endophytic Bacillus megaterium colonization on structure strengthening, microbial community, chemical composition and stabilization properties of Hybrid Pennisetum. *J. Sci. Food Agric.* 100 1164–1173. 10.1002/jsfa.10125 31680258

[B48] Shah A. A. Wu J. Qian C. Liu Z. Mobashar M. Tao Z. (2020b). Ensiling of whole-plant hybrid pennisetum with natamycin and Lactobacillus plantarum impacts on fermentation characteristics and meta-genomic microbial community at low temperature. *J. Sci. Food Agric.* 100 3378–3385. 10.1002/jsfa.10371 32144784

[B49] Shen X. Gong X. Cai Y. Guo Y. Tu J. Li H. (2016). Normalization and integration of large-scale metabolomics data using support vector regression. *Metabolomics* 12:89. 10.1007/s11306-016-1026-5

[B50] Song X. Yue X. Chen W. Jiang H. Han Y. Li X. (2019). Detection of cadmium risk to the photosynthetic performance of *Hybrid Pennisetum*. *Front. Plant Sci.* 10:798. 10.3389/fpls.2019.00798 31281328 PMC6596316

[B51] Soni R. Keharia H. (2021). Phytostimulation and biocontrol potential of Gram-positive endospore-forming Bacilli. *Planta* 254:49. 10.1007/s00425-021-03695-0 34383174

[B52] Suaisom P. Pholchan P. Aggarangsi P. (2019). Holistic determination of suitable conditions for biogas production from *Pennisetum purpureum* × *Pennisetum americanum* liquor in anaerobic baffled reactor. *J. Environ. Manage.* 247 730–737. 10.1016/j.jenvman.2019.06.103 31279804

[B53] Trostchansky A. Wood I. Rubbo H. (2021). Regulation of arachidonic acid oxidation and metabolism by lipid electrophiles. *Prostaglandins Other Lipid Mediat.* 152:106482. 10.1016/j.prostaglandins.2020.106482 33007446

[B54] Urbanek A. K. Rybak J. Wróbel M. Leluk K. Mirończuk A. M. (2020). A comprehensive assessment of microbiome diversity in *Tenebrio molitor* fed with polystyrene waste. *Environ. Pollut.* 262:114281. 10.1016/j.envpol.2020.114281 32146369

[B55] Van Soest P. J. Robertson J. B. Lewis B. A. (1991). Methods for dietary fiber, neutral detergent fiber, and nonstarch polysaccharides in relation to animal nutrition. *J. Dairy Sci.* 74 3583–3597. 10.3168/jds.S0022-0302(91)78551-2 1660498

[B56] Wang B. Gao R. Wu Z. Yu Z. (2020). Functional analysis of sugars in modulating bacterial communities and metabolomics profiles of *Medicago sativa* silage. *Front. Microbiol.* 11:641. 10.3389/fmicb.2020.00641 32477276 PMC7232540

[B57] Wang C. Zheng M. Wu S. Zou X. Chen X. Ge L. (2021). Effects of gallic acid on fermentation parameters, protein fraction, and bacterial community of whole plant soybean silage. *Front. Microbiol.* 12:662966. 10.3389/fmicb.2021.662966 34079531 PMC8165322

[B58] Wang D. Guo J.-R. Liu X.-J. Song J. Chen M. Wang B.-S. (2014). Effects of cultivation strategies on hybrid *Pennisetum* yield in saline soil. *Crop Sci.* 54 2772–2781. 10.2135/cropsci2013.11.0741 34798789

[B59] Wang Y. Guo X. Li K. Nan Y. Wang J. Zhang J. (2019). Comparison of a solvent mixture assisted dilute acid and alkali pretreatment in sugar production from hybrid *Pennisetum*. *Ind. Crops Prod.* 141:111806.

[B60] Wang M. Wang L. Yu Z. (2019). Fermentation dynamics and bacterial diversity of mixed lucerne and sweet corn stalk silage ensiled at six ratios. *Grass Forage Sci.* 74 264–273. 10.1111/gfs.12431

[B61] Xu D. Ding W. Ke W. Li F. Zhang P. Guo X. (2019). Modulation of metabolome and bacterial community in whole crop corn silage by inoculating homofermentative *Lactobacillus plantarum* and heterofermentative *Lactobacillus buchneri*. *Front. Microbiol.* 9:3299. 10.3389/fmicb.2018.03299 30728817 PMC6352740

[B62] Xu D. Ding Z. Wang M. Bai J. Ke W. Zhang Y. (2020). Characterization of the microbial community, metabolome and biotransformation of phenolic compounds of sainfoin (*Onobrychis viciifolia*) silage ensiled with or without inoculation of *Lactobacillus plantarum*. *Bioresour. Technol.* 316:123910. 10.1016/j.biortech.2020.123910 32750640

[B63] Xu D. Wang N. Rinne M. Ke W. Weinberg Z. G. Da M. (2021). The bacterial community and metabolome dynamics and their interactions modulate fermentation process of whole crop corn silage prepared with or without inoculants. *Microb. Biotechnol.* 14 561–576. 10.1111/1751-7915.13623 32627363 PMC7936295

[B64] Yuan X. Dong Z. Liu J. Shao T. (2020). Microbial community dynamics and their contributions to organic acid production during the early stage of the ensiling of Napier grass (*Pennisetum purpureum*). *Grass Forage Sci.* 75 37–44.

[B65] Yuan X. Wen A. Dong Z. Desta S. T. Shao T. (2017). Effects of formic acid and potassium diformate on the fermentation quality, chemical composition and aerobic stability of alfalfa silage. *Grass Forage Sci.* 72 833–839. 10.1002/jsfa.8475 28585343

[B66] Zhang Q. Guo X. Zheng M. Chen D. Chen X. (2021). Altering microbial communities: a possible way of lactic acid bacteria inoculants changing smell of silage. *Anim. Feed Sci. Technol.* 279:114998. 10.1016/j.anifeedsci.2021.114998

[B67] Zhao J. Xia B. Meng Y. Yang Z. Pan L. Zhou M. (2019). Transcriptome analysis to shed light on the molecular mechanisms of early responses to cadmium in roots and leaves of king grass (*Pennisetum americanum* × *P. purpureum)*. *Int. J. Mol. Sci.* 20:2532. 10.3390/ijms20102532 31126029 PMC6567004

[B68] Zi X. Li M. Chen Y. Lv R. Zhou H. Tang J. (2021). Effects of citric acid and *Lactobacillus plantarum* on silage quality and bacterial diversity of king grass silage. *Front. Microbiol.* 12:631096. 10.3389/fmicb.2021.631096 33717021 PMC7953137

[B69] Zieliński M. Rusanowska P. Zielińska M. Dudek M. Nowicka A. Purwin C. (2021). Influence of preparation of Sida hermaphrodita silages on its conversion to methane. *Renew. Energy* 163 437–444. 10.1016/j.renene.2020.09.012

