# Supplementary material for: Additives Altered Bacterial Communities and Metabolic Profiles in Silage Hybrid Pennisetum
Source: Front Microbiol. 2022 Jan 5;12:770728. doi: 10.3389/fmicb.2021.770728 (PMC8767026; doi:10.3389/fmicb.2021.770728)
Supplement: Supplementary file 1 [file Table_1.DOCX]

**Table S1** Effects of silage additives on the α-diversity of bacterial community in silage hybrid *Pennisetum*

|  | CK | MA | GL | CE | BS |
| --- | --- | --- | --- | --- | --- |
| Observed species | 778.78±142.69^b^ | 1321.33±222.22^a^ | 754.98±139.59^b^ | 854.45±415.50^b^ | 879.30±243.93^b^ |
| Faith_PD | 65.14±11.49^b^ | 85.86±10.22^a^ | 61.53±7.46^b^ | 67.66±20.92^b^ | 69.61±13.76^ab^ |
| Pielou evenness | 0.46±0.03^b^ | 0.57±0.05^a^ | 0.44±0.05^bc^ | 0.40±0.04^c^ | 0.47±0.05^b^ |
| Chao1 index | 877.76±158.53^b^ | 1485.57±211.82^a^ | 848.86±174.17^b^ | 968.03±447.85^b^ | 1027.88±323.32^b^ |
| Shannon index | 4.45±0.40^b^ | 5.91±0.62^a^ | 4.18±0.54^bc^ | 3.85±0.59^c^ | 4.55±0.61^b^ |
| Simpson index | 0.83±0.04^b^ | 0.92±0.03^a^ | 0.81±0.07^bc^ | 0.75±0.06^c^ | 0.83±0.06^b^ |

CK, control group; MA, 1% FM malic acid addition; GL, 1% FM glucose addition; CE, 100 U/g FM cellulase addition; BS, 10^6^ cfu/g *Bacillus subtilis* FM addition. DM, dry matter; FM, fresh matter.
