# Supplementary material for: Additives Altered Bacterial Communities and Metabolic Profiles in Silage Hybrid Pennisetum
Source: Front Microbiol. 2022 Jan 5;12:770728. doi: 10.3389/fmicb.2021.770728 (PMC8767026; doi:10.3389/fmicb.2021.770728)
Supplement: Supplementary file 2 [file Table_2.DOCX]

**Table S2** Effects of silage additives on the dominant phyla and genera in silage hybrid *Pennisetum*

| Items | Treatments^1^ | | | | | SEM^2^ | P value |
| --- | --- | --- | --- | --- | --- | --- | --- |
|  | CK | MA | GL | CE | BS |  |  |
| Phyla (Relative abundance) | | | | | | | |
| *Proteobacteria* | 0.7046^ab^ | 0.3243^c^ | 0.5534^b^ | 0.7676^a^ | 0.5591^b^ | 0.0385 | 0.0002 |
| *Firmicutes* | 0.2718^bc^ | 0.6395^a^ | 0.4270^b^ | 0.2093^c^ | 0.4124^b^ | 0.0373 | 0.0002 |
| *Bacteroidetes* | 0.0139^ab^ | 0.0184^a^ | 0.0064^b^ | 0.0152^ab^ | 0.0114^ab^ | 0.0018 | 0.3359 |
| *Actinobacteria* | 0.0036^b^ | 0.0102^a^ | 0.0074^ab^ | 0.0033^b^ | 0.0073^ab^ | 0.0008 | 0.0081 |
| *Deinococcus-Thermus* | 0.0032^b^ | 0.0035^b^ | 0.0036^b^ | 0.0024^b^ | 0.0071^a^ | 0.0005 | 0.0264 |
| *Spirochaetes* | 0.0008 | 0.0009 | 0.0004 | 0.0005 | 0.0005 | 0.0001 | 0.6894 |
| *Tenericutes* | 0.0003^b^ | 0.0008^a^ | 0.0002^b^ | 0.0006^ab^ | 0.0005^ab^ | 0.0001 | 0.0271 |
| *Chloroflexi* | 0.0002 | 0.0002 | 0.0002 | 0.0001 | 0.0003 | 0.0001 | 0.8728 |
| *Acidobacteria* | 0.0003 | 0.0001 | 0.0002 | 0.0001 | 0.0002 | 0.0000 | 0.5780 |
| *Patescibacteria* | 0.0003 | 0.0001 | 0.0002 | 0.0002 | 0.0001 | 0.0000 | 0.6907 |
| Genera (Relative abundance) | | | | | | | |
| *Aquabacterium* | 0.5435^ab^ | 0.2699^c^ | 0.4825^b^ | 0.6741^a^ | 0.5019^b^ | 0.0346 | 0.0006 |
| *Bacillus* | 0.0335^c^ | 0.3540^a^ | 0.0403^c^ | 0.0193^c^ | 0.1124^b^ | 0.0254 | 0.0000 |
| *Weissella* | 0.1003^a^ | 0.0151^b^ | 0.1822^a^ | 0.0776^ab^ | 0.1403^a^ | 0.0192 | 0.0594 |
| *Lactobacillus* | 0.0744 | 0.0479 | 0.0511 | 0.0408 | 0.0432 | 0.0066 | 0.4906 |
| *Brevibacillus* | 0.0029^b^ | 0.0266^a^ | 0.0196^ab^ | 0.0054^b^ | 0.0172^ab^ | 0.0030 | 0.0240 |
| *Paenibacillus* | 0.0041 | 0.0384 | 0.0678 | 0.0027 | 0.0070 | 0.0111 | 0.3104 |
| *Novosphingobium* | 0.0282^a^ | 0.0040^c^ | 0.0148^bc^ | 0.0172^ab^ | 0.0061^bc^ | 0.0024 | 0.0031 |
| *Acinetobacter* | 0.0202^a^ | 0.0181^ab^ | 0.0021^c^ | 0.0078^ac^ | 0.0045^bc^ | 0.0025 | 0.0541 |
| *Pelomonas* | 0.0136^a^ | 0.0081^ab^ | 0.0043^b^ | 0.0096^ab^ | 0.0112^ab^ | 0.0012 | 0.2387 |
| *Un_Burkholderiaceae* | 0.0162^a^ | 0.0060^b^ | 0.0077^b^ | 0.0112^ab^ | 0.0068^b^ | 0.0010 | 0.0030 |

^1^ CK, control group; MA, 1% FM malic acid addition; GL, 1% FM glucose addition; CE, 100 U/g FM cellulase addition; BS, 10^6^ cfu/g *Bacillus subtilis* FM addition. DM, dry matter; FM, fresh matter.

^2^ SEM, standard error of means.
