# Supplementary material for: Additives Altered Bacterial Communities and Metabolic Profiles in Silage Hybrid Pennisetum
Source: Front Microbiol. 2022 Jan 5;12:770728. doi: 10.3389/fmicb.2021.770728 (PMC8767026; doi:10.3389/fmicb.2021.770728)
Supplement: Supplementary file 4 [file Table_4.DOCX]

**Table S4** Effects of silage additives on the dominant functions and pathways of bacterial communities predicted by PICRUSt in silage hybrid Pennisetum.

|  | CK | MA | GL | CE | BS |
| --- | --- | --- | --- | --- | --- |
| Functions (Relative abundance) | | | | | |
| DNA helicase | 0.0122 | 0.0118 | 0.0128 | 0.0121 | 0.0124 |
| DNA-directed DNA polymerase | 0.0121 | 0.0115 | 0.0125 | 0.0121 | 0.0121 |
| Histidine kinase | 0.0106 | 0.0109 | 0.0103 | 0.0109 | 0.0105 |
| NADH: ubiquinone reductase (H(+)-translocating) | 0.0094 | 0.0063 | 0.0085 | 0.0102 | 0.0083 |
| Peptidylprolyl isomerase | 0.0089 | 0.0058 | 0.0083 | 0.0095 | 0.0080 |
| Acetyl-CoA C-acetyltransferase | 0.0057 | 0.0042 | 0.0051 | 0.0061 | 0.0051 |
| DNA-directed RNA polymerase | 0.0050 | 0.0051 | 0.0052 | 0.0049 | 0.0051 |
| 3-oxoacyl-[acyl-carrier-protein] reductase | 0.0045 | 0.0057 | 0.0047 | 0.0046 | 0.0048 |
| Cytochrome-c oxidase | 0.0046 | 0.0047 | 0.0045 | 0.0050 | 0.0046 |
| Protein-N(pi)-phosphohistidine --- sugar phosphotransferase | 0.0039 | 0.0060 | 0.0046 | 0.0033 | 0.0047 |
| Pathways (Relative abundance) | | | | | |
| Aerobic respiration I (cytochrome c) | 0.0141 | 0.0135 | 0.0131 | 0.0154 | 0.0134 |
| Gondoate biosynthesis (anaerobic) | 0.0083 | 0.0078 | 0.0087 | 0.0081 | 0.0083 |
| CDP-diacylglycerol biosynthesis I | 0.0079 | 0.0069 | 0.0080 | 0.0080 | 0.0076 |
| CDP-diacylglycerol biosynthesis II | 0.0079 | 0.0069 | 0.0080 | 0.0080 | 0.0076 |
| Cis-vaccenate biosynthesis | 0.0075 | 0.0074 | 0.0081 | 0.0075 | 0.0077 |
| Fatty acid elongation --- saturated | 0.0077 | 0.0077 | 0.0075 | 0.0076 | 0.0077 |
| Fatty acid beta-oxidation I | 0.0079 | 0.0068 | 0.0070 | 0.0086 | 0.0073 |
| Pyruvate fermentation to isobutanol (engineered) | 0.0080 | 0.0079 | 0.0070 | 0.0077 | 0.0070 |
| Superpathway of adenosine nucleotides de novo biosynthesis I | 0.0074 | 0.0067 | 0.0077 | 0.0075 | 0.0073 |
| Superpathway of pyrimidine nucleobases salvage | 0.0072 | 0.0069 | 0.0077 | 0.0072 | 0.0072 |

CK, control group; MA, 1% FM malic acid addition; GL, 1% FM glucose addition; CE, 100 U/g FM cellulase addition; BS, 10^6^ cfu/g *Bacillus subtilis* FM addition. DM, dry matter; FM, fresh matter.
