# Supplementary material for: Additives Altered Bacterial Communities and Metabolic Profiles in Silage Hybrid Pennisetum
Source: Front Microbiol. 2022 Jan 5;12:770728. doi: 10.3389/fmicb.2021.770728 (PMC8767026; doi:10.3389/fmicb.2021.770728)
Supplement: Supplementary file 6 [file Table_6.DOCX]

**Table S6** Nine pathways affected by all the four additives in silage hybrid *Pennisetum*

| Pathways  (Relative abundance) | Treatments^1^ | | | | | SEM^2^ | P value |
| --- | --- | --- | --- | --- | --- | --- | --- |
|  | CK | MA | GL | CE | BS |  |  |
| Superpathway of thiamin diphosphate biosynthesis II | 7.40E-04 | 2.62E-03 | 1.17E-03 | 2.80E-03 | 1.95E-03 | 1.81E-04 | 1.97E-06 |
| PpGpp biosynthesis | 1.05E-03 | 4.53E-04 | 7.31E-04 | 4.91E-04 | 5.58E-04 | 5.38E-05 | 1.24E-04 |
| Syringate degradation | 7.84E-04 | 2.75E-04 | 3.26E-04 | 3.53E-04 | 4.42E-04 | 4.67E-05 | 3.86E-04 |
| Protocatechuate degradation I (meta-cleavage pathway) | 6.18E-04 | 1.91E-04 | 4.04E-04 | 2.51E-04 | 3.29E-04 | 3.57E-05 | 4.41E-05 |
| Methylgallate degradation | 5.85E-04 | 1.84E-04 | 3.83E-04 | 2.43E-04 | 3.12E-04 | 3.35E-05 | 4.89E-05 |
| Gallate degradation II | 4.92E-04 | 1.49E-04 | 3.22E-04 | 1.98E-04 | 2.56E-04 | 2.90E-05 | 6.53E-05 |
| Superpathway of salicylate degradation | 2.85E-04 | 1.45E-04 | 1.66E-04 | 1.23E-04 | 1.80E-04 | 1.38E-05 | 1.01E-04 |
| Toluene degradation IV (aerobic) (via catechol) | 1.29E-04 | 8.13E-05 | 5.21E-05 | 6.17E-05 | 4.18E-05 | 7.63E-06 | 2.45E-04 |
| Nitrate reduction I (denitrification) | 4.31E-05 | 1.92E-05 | 2.00E-05 | 1.78E-05 | 1.74E-05 | 2.57E-06 | 7.31E-04 |

^1^ CK, control group; MA, 1% FM malic acid addition; GL, 1% FM glucose addition; CE, 100 U/g FM cellulase addition; BS, 10^6^ cfu/g *Bacillus subtilis* FM addition. DM, dry matter; FM, fresh matter.

^2^ SEM, standard error of means.
