# Supplementary material for: Additives Altered Bacterial Communities and Metabolic Profiles in Silage Hybrid Pennisetum
Source: Front Microbiol. 2022 Jan 5;12:770728. doi: 10.3389/fmicb.2021.770728 (PMC8767026; doi:10.3389/fmicb.2021.770728)

**Fig.S1** Relative standard deviation of QC samples. (a) Positive ion mode; (b) Negative ion mode


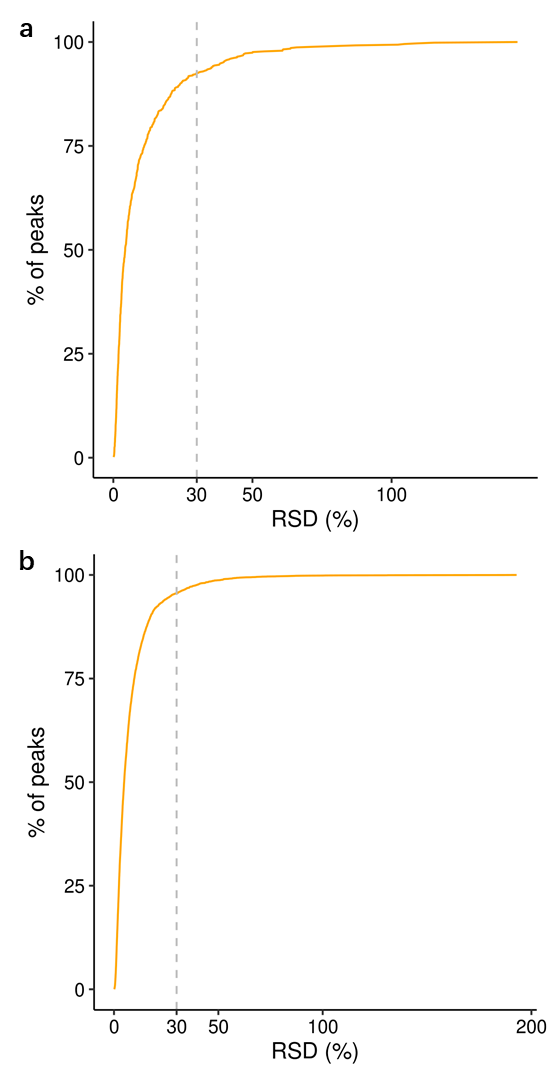


**Fig.S2** Principal component analysis of experimental samples and QC samples. (a) Positive ion mode; (b) Negative ion mode


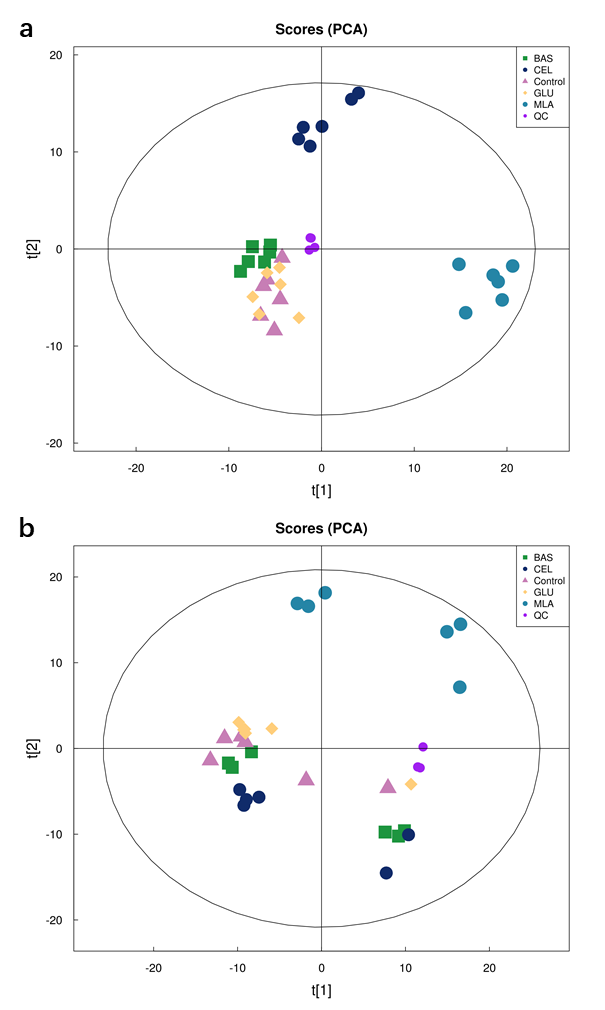


**Fig.S3** Superclass of differential metabolites


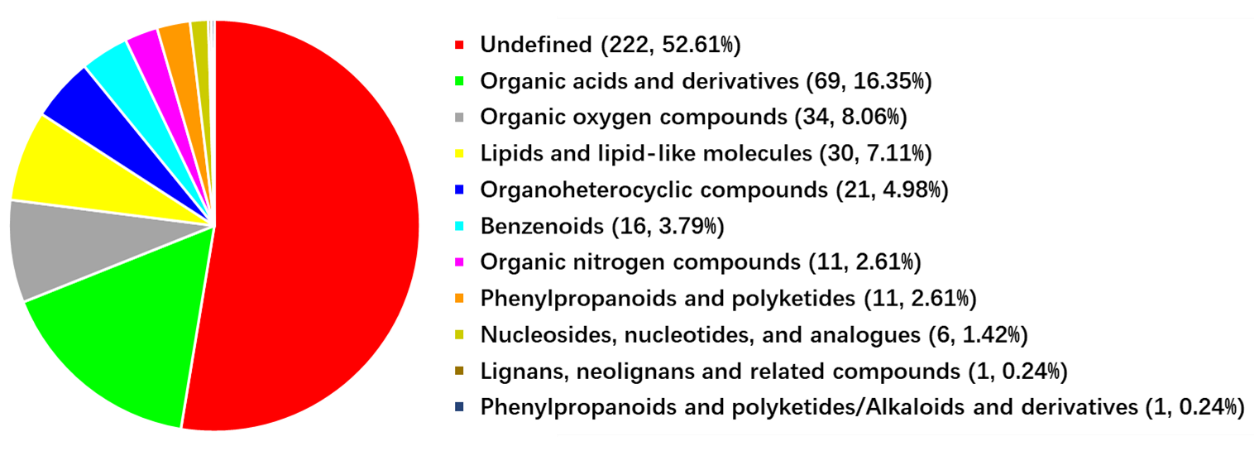

Supplement: Supplementary file 10 [file Data_Sheet_1.docx]
